# Supplementary material for: Ubiquitous purine sensor modulates diverse signal transduction pathways in bacteria
Source: Nat Commun. 2024 Jul 12;15:5867. doi: 10.1038/s41467-024-50275-3 (PMC11245519; doi:10.1038/s41467-024-50275-3)
Supplement: Supplementary file 1 — Supplementary Information [file 41467_2024_50275_MOESM1_ESM.pdf]

Supplementary material

to

Ubiquitous purine sensor modulates diverse signal transduction pathways in bacteria

by

Elizabet Monteagudo-Cascales, Vadim M. Gumerov, Matilde Fernández, Miguel A. Matilla, José A. Gavira, Igor B. Zhulin and Tino Krell

Supplementary Fig. 1. Representative section of the composite |2Fo-Fc| omit map of the McpH-LBD structure contoured at 1.5 σ. The bound ligand, uric acid, is shown in the centre.

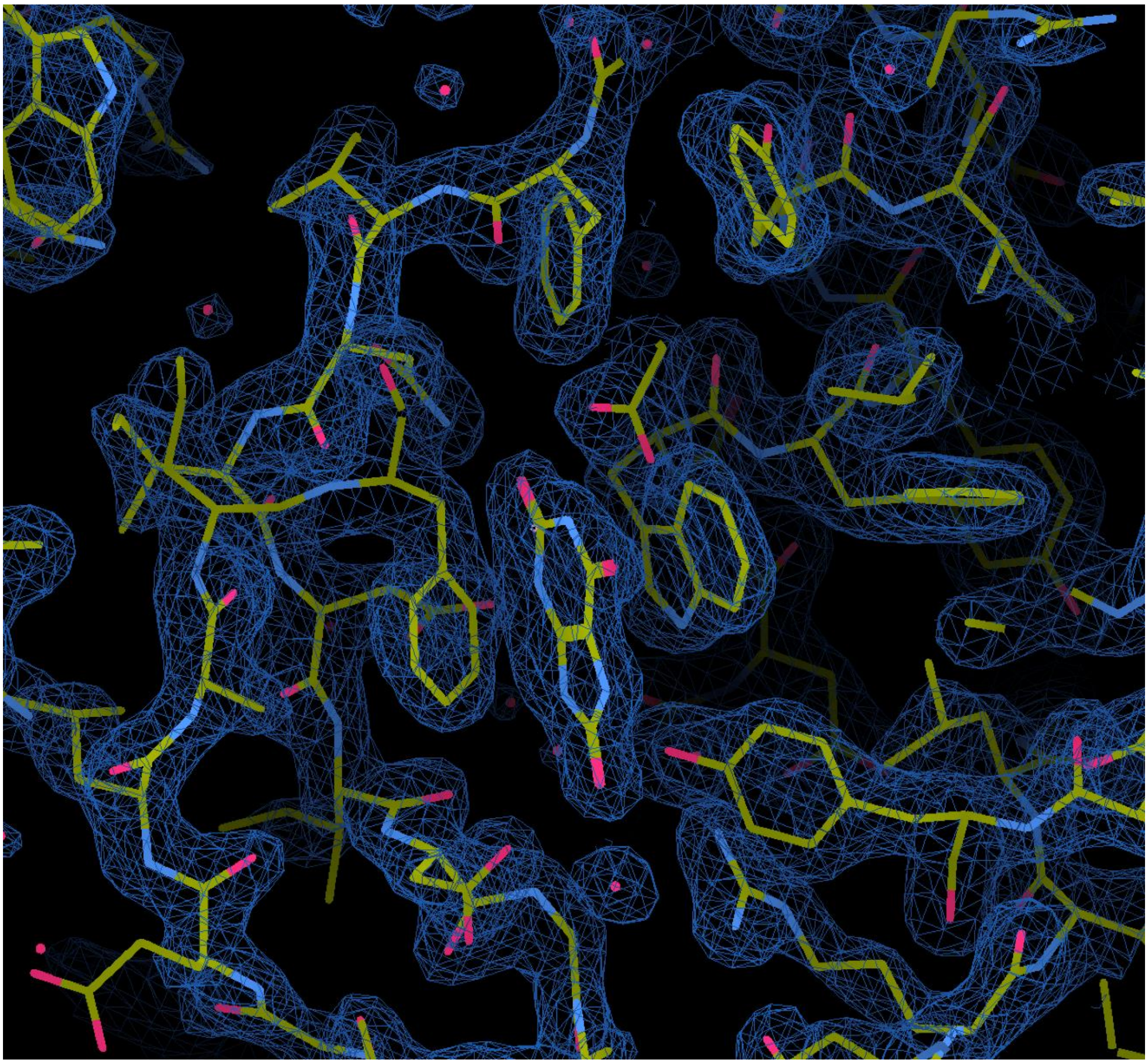

Supplementary Figure 2. Schematic representation of the the molecular detail of uric acid recognition by McpH-LBD. Hydrogen bonds are shown as dotted lines and distances are indicated in Å. Hydrophobic interactions are shown as spiked arks. The amino acids that are part of the purine binding motif are labelled in red. The interactions were identified by Ligplot that uses a cut-off of 3.0 Å[1].

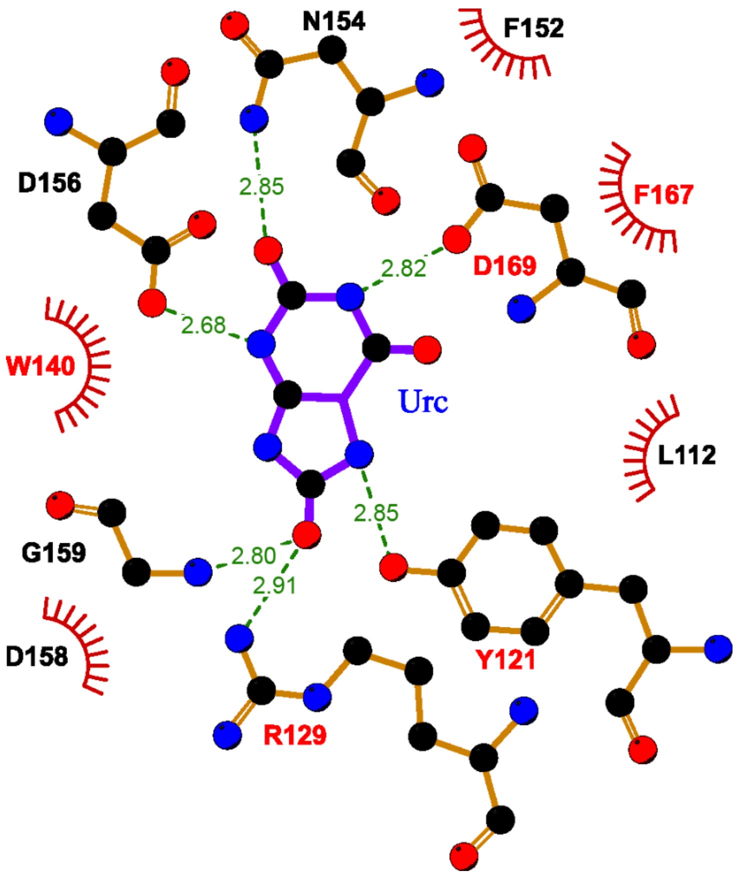

Supplementary Figure 3. Multiple sequence alignment of amino acid, amine and purine binding dCache_1 domains. Ligand-binding motifs are shown: purine motif (cyan), amine motif (green), amino acid motif (grey). Gproteobac: Gammaproteobacteria. Extensions R1, R2 etc. mark the proteins that have been analyzed in this study (Table 2).

```
AA_Proteobacteria_NP_252999.1           ---------------YL-------QRNAIREDLESYLREMGDVTSSN-IQNWLGGRLLLV
AA_Cyanobacteria_WP_162544314.1         --------------------NDIVMREQTEERVNSNLSMAGSSVAKS-VSNWLTGRTMLT
AA_Actinobacteriota_WP_187050447.1      --------------------SYKNSIELTTQEITGSTEEQVKSMNDS-FETFLQKTENQL
AA_Bacteroidota_WP_196119247.1          ----------------------NDSKKIVVNQIKLNNYETLQNVNDYFLKNFMYDMEYII
AA_Thermotogota_WP_114702178.1          ----G------------------------KELGTTSLLSIATSGTKN-LENFLQGYVNLV
AA_Methanobacteriota_WP_056934003.1     -------------LNKMDEAMSKGMEKPVIDNGKLLATNAANLAARM-FDDYFSSIADYG
AA_Firmicutes_NP_389278.2               -------------------------KPMITEDGKNTTQNVTQSLEQN-IELQLKSYAISL
AA_Desulfobacterota_WP_144684422.1      -------------------------RKNSVENFHTGTAKELTHIEAA-MDIFMENASNIT
AA_Chloroflexota_WP_116224856.1         ------------------------YKSKQLETVQTSIDSQLALLDFS-LTNFITEVKNNV
AA_Halobacterota_WP_004039109.1         ------------------------LHEQNLDAISDNTELQLRHIEFA-LTNFISSAHYDV
AA_Thermoplasmatota_OPX61375.1          ------------------------LEDQELDTVENESITQLEHIDFA-LTNMIEESKADI
AA_Spirochaetota_WP_018527208.1         ------------------------SRTLSNRLIFQGLRQDLASLQQS-VESWAQKKLVLI
AA_Singergistota_WP_012869308.1         -------------------------HSILNDQVNTLGMEMIANNANQ-VDQYFSQLKTLT
AA_Verrucomicrobiota_WP_176014353.1     RSLVGLNAKALAQCQAIGDNAVRDSRTALIGMARTHLRDFATDQAAI-TDVKFKHIAAEV
AA_Planctomycetota_MBG80894.1           --------------------QFDTLRDTAMANARQRAQLVSESWASH-FNGQLHGIAQAV
AA_Campylobacterota_WP_010891944.1      -------------------------KTSLYESTLKNQTDLLKVTQST-VEDFRSTNQSFT
AA_Chrysiogenetota_WP_183731730.1       ----------------M-------KNHMLQQQLKNTEDLLTTVASSS-ITPWVNTRLRYI
AA_Acidobacteriota_HCZ33114.1           ---------------------------SQQQALERQAGARAQAALRH-LDEALRSDLEAS
AA_Deinococcota_WP_184108923.1          ------------------WNDRQNAERVVQAEAETTLGRLAQVAAEN-IRASLQSPTQLV
AA_Thermodesulfobacteria_RUM89741.1     -------------------LNVYQYFKIKPHFASEIMARLNRTEVNN-IKGFFAQVSEKL
AA_Fibrobacterota_WP_022637451.1        ------------------HTQHMDYDSLLTQQGKNTIHGITEHTALR-ISELLKEPALV-
AM_Desulfobacterota_WP_027185430.1      ---------------------HTSSKTTLMQEARADAANLTLASIRK-IEGTLASVEAIP
AM_Aproteobac_McpX_WP_014528895.1       --------------------LISQTQDRVETLVFDGAKTEARAIASD-IAGSVGELAAAA
AM_Gproteobac_PacA_WP_039291840.1       --------------------------------AERYLQQIAQSEALR-IQQELNYARDVA
AM_Halobacteriota_WP_011034866.1        -------------------------TTQEEKLAYQQSVEMASNYANQ-FDADMKANLAIA
AM_Spirochaetota_WP_015707342.1         ---------------------SNRSIEMAQKDAFSLAQETADKYKNA-IIAELQGARITA
AM_Verrucomicrobiota_WP_129046516.1     -----------------------RVVHSARQEANALSRTKAQAIGAE-MAHRLGRAIGTA
AM_Aproteobac_WP_041812266.1            ---------------------TTKSGSDIETLAFQSGEQLGHRYGEM-VHARLGNAMEAG
AM_Bacteroidota_WP_092437134.1          ---------------------TYNSLKTATVSSTEISNQMAVTYANQ-VVDKMDDAMSAA
AM_Firmicutes_WP_056043800.1            --------------------QLMKLYDVSLRQGELVAQNQSNAYTTK-MSIETNDALIRL
AM_Firmicutes_WP_052635864.1            --------------------SYINARNEALNAAQKRAQIVAKNYSKE-ISDELGQAITVA
AM_Gproteobac_PctD_WP_038403940.1       ------------------------------KVINERLVALARAQVSQ-IQRELEYPLTVV
PU_Gproteobac_R3_WP_124259872.1         ----------------------------KERAREQDLPTALGEIRSE-VLRQIAAPVALT
PU_Gproteobac_McpH_WP_047603170.1       ------------------------NRLTDRYLVDTALPASIEAIRND-IERMLGQPLVAA
PU_Gproteobac_R1_WP_219614703.1         --------------------QRSAQQLIETRMFEQELPNLTQRIGKE-IEKDLTSVANAA
PU_Firmicutes_A_R5_WP_131005693.1       --------------------GYQSNTNIFKNDIEHVSTLAAEGIYYQ-IDKLLSEPINVS
PU_Actinobacteriota_R8_WP_227113344.1   --------------------GYQSSRAAFEKDAERTSLLAAEGAARE-IDNRFAEPIDVS
PU_Cyanobacteria_R9_WP_198537540.1      ---------------------ANALAAARRQVVESTLPLTLDALSAD-LQQDFVQPILFA
PU_Gproteobac_R7_WP_038903150.1         ------------------------RHSLFDEISESSLPLTSDNVYSE-IQRDLLNPIFIS
PU_Gproteobac_R6_WP_185834821.1         ------------------------HDTLEEQINKDSLPLTSDNIYSE-IQQDLIRPIFIS
PU_Verrucomicrobiota_R10_WP_110129388.1 -------------------V---SRNNVRKTLAESTLPLTSDNVYSE-IQRDLLRPVFIA
PU_Verrucomicrob_R11_WP_162024566.1     ------------------------RSNMRLSITESTLPLTSDNVYSE-IQRDLLRPIFIS
PU_Verrucomicrobiota_R12_WP_220621668.1 -----------------------------------SLPLTADNIYSV-IQRDLLRPIFIS
PU_Campylobacterota_R2_WP_107944080.1   -------------------------NLYTEKVVKDELPLAVSNVAGE-IGYAIDKIINTS
PU_Firmicutes_R13_WP_167859577.1        -----------------------TQREAVDKLKTKDLLHLADSIAAK-VDGQIRKAKETS
PU_Firmicutes_R15_WP_207952809.1        ------------------------EKEVVNKLKSKDLVRIAESIASK-IDGRLQRAQESS
PU_Firmicutes_R14_WP_021170906.1        -----------------------THNAMVDKLKNRDMLYIVQSMSEK-IDGRIERAQETS
PU_Gproteobac_R4_WP_199478134.1         ------------------------MESDLKQLKEELLPNRLHSLSSR-ISEQISPLINAS

AA_Proteobacteria_NP_252999.1           E---QTAQTLAR-----------------------------------------DHSPETV
AA_Cyanobacteria_WP_162544314.1         E---FAMQSLGA-----------------------------------------KPASEMT
AA_Actinobacteriota_WP_187050447.1      D---RISKYPIMSTY--------------------------------------DKNPESI
AA_Bacteroidota_WP_196119247.1          N---DWASKDDLKNY--------------------------------RNHANQLKMVTAI
AA_Thermotogota_WP_114702178.1          D---FLSSDANVV----------------------------------GAKENKYDEVTWM
AA_Methanobacteriota_WP_056934003.1     H----MADFAVQEAY---------------------------------KNGLKGDELRQYL
AA_Firmicutes_NP_389278.2               S---RLANGELTHTFVTKP---------------------------------SKEASRLF
AA_Desulfobacterota_WP_144684422.1      R---MLAMHPEVKKIDEN-----------------LSSHTRTTRPTRPSDMTPGETEEKI
AA_Chloroflexota_WP_116224856.1         L---ALSENEIIRTQEDHD----------------FTNFLEADEE--TFEYHIGDVEQNI
AA_Halobacterota_WP_004039109.1         H---ELSLNEIVRNPDDAD----------------FTNFLNASEE--TYQYSITDEEQAI
AA_Thermoplasmatota_OPX61375.1          L---QLSMDERVQCRDDSN----------------FTNFLNATAE--DFEYNITPEEQAI
AA_Spirochaetota_WP_018527208.1         D---SVGHTLET---------------------------------------------PGM
```

```
AA_Singergistota_WP_012869308.1         L---GIASGTAELLET-------------------------------GQAVTDDDLESVM
AA_Verrucomicrobiota_WP_176014353.1     ESAAALSRELMARPHAGGNVLLYSAVNTPPLDKRQYSSLYFAPEVATETVQAAMPPLSQL
AA_Planctomycetota_MBG80894.1           D---AMAA---------------------------------------AVGQESVRNEDSL
AA_Campylobacterota_WP_010891944.1      R---ALEKDIANLPYQ------------------------------------SLITEENI
AA_Chrysiogenetota_WP_183731730.1       D---TLSYEDDL-----------------------------------LTLALERQATDTL
AA_Acidobacteriota_HCZ33114.1           Q---SLGQVVQSWW---------------------------------LEGVLEPTRPERA
AA_Deinococcota_WP_184108923.1          D-------------ITTELI---------------------------RTGQLDTADATRL
AA_Thermodesulfobacteria_RUM89741.1     LTVHDLGKNGVL----------------------------------------KFEDIINL
AA_Fibrobacterota_WP_022637451.1        ----NYTNKTYIR----------------------------------MQSLYDENDLSPL
AM_Desulfobacterota_WP_027185430.1      G---LLAFSYGK----------------------------------------NKPTASAI
AM_Aproteobac_McpX_WP_014528895.1       R---TMSGVLGR------------------------------------GHAGQSTDRAGA
AM_Gproteobac_PacA_WP_039291840.1       H---NLGQGLAA------------------------------------LPSAGIKDRAVV
AM_Halobacteriota_WP_011034866.1        R---TISTTMES---------------------------------------YETADRDEA
AM_Spirochaetota_WP_015707342.1         E---TFSTVFET------------------------------------LKDYNLTDRDMM
AM_Verrucomicrobiota_WP_129046516.1     R---TLSEALEG------------------------------------ILAEGHPSRAQA
AM_Aproteobac_WP_041812266.1            R---FIATSLVG------------------------------------LKAAGRTDREQL
AM_Bacteroidota_WP_092437134.1          R---SLAHALSG-VIG------------------------------------KNVSRQAI
AM_Firmicutes_WP_056043800.1            E---GLQQSLQQ------------------------------------MKEYNMTDRSEA
AM_Firmicutes_WP_052635864.1            E---NLGSMLKGR----------------------------------IRNENATLTRDEV
AM_Gproteobac_PctD_WP_038403940.1       H---GLANSTRLLGEPGA-----------------------------DGMPQLNASRDEI
PU_Gproteobac_R3_WP_124259872.1         R---SLATNEYILNWE-------------------------------EQGL-PEQGAAAW
PU_Gproteobac_McpH_WP_047603170.1       A---DIAGNTLLRDWL-------------------------------AAGE-DPAQAPQF
PU_Gproteobac_R1_WP_219614703.1         R---QLANDRFVLDWV-------------------------------ARGM-PKEQESIL
PU_Firmicutes_A_R5_WP_131005693.1       L---TMANDSLLKNFLDGE----------------------------KEHLNDEEFIYKL
PU_Actinobacteriota_R8_WP_227113344.1   L---AMAHDTLLADLLSAE----------------------------PTRGDDEAYADAV
PU_Cyanobacteria_R9_WP_198537540.1      S---AMAANTLLIDWV-------------------------------EQG---EQPEAAV
PU_Gproteobac_R7_WP_038903150.1         S---LMAHDTFVKDWV-------------------------------LSN---ETDPQAM
PU_Gproteobac_R6_WP_185834821.1         S---LMAQDTFVREWT-------------------------------LAG---EQDPERI
PU_Verrucomicrobiota_R10_WP_110129388.1 S---LMANDTFLRDWA-------------------------------IAG---EKDRDAI
PU_Verrucomicrob_R11_WP_162024566.1     S---LMANDTFLRDWA-------------------------------LNG---EVDINQI
PU_Verrucomicrobiota_R12_WP_220621668.1 S---MMANDAFLRDWT-------------------------------ING---EVDVKQM
PU_Campylobacterota_R2_WP_107944080.1   Y---QMTKNDYLLKWI-------------------------------DEGE-PKDGLATL
PU_Firmicutes_R13_WP_167859577.1        L---LMAEDPNVINWV-------------------------------AGGERDEALGAVV
PU_Firmicutes_R15_WP_207952809.1        L---TMAMDPELIEWL-------------------------------ASGEKDQIAGAHV
PU_Firmicutes_R14_WP_021170906.1        L---LLADDPTVLAWV-------------------------------ESGGRDEAAGEIV
PU_Gproteobac_R4_WP_199478134.1         K---LMTNDRFIADWV-------------------------------KKGA-DESRLPLV

AA_Proteobacteria_NP_252999.1           SALLEQ--------------PALTSTFSFTYLGQ--------------------------
AA_Cyanobacteria_WP_162544314.1         PEQFNV--------------PLLTSTFLMTYYGN--------------------------
AA_Actinobacteriota_WP_187050447.1      MDEFSHT-------------QSSSSEINGLYLGTE-------------------------
AA_Bacteroidota_WP_196119247.1          PKHFKPISD------QWTGYVNSIPDIAWIYLGVE-------------------------
AA_Thermotogota_WP_114702178.1          LKTFKNI-------------KDKYKDALWIYLGT--------------------------
AA_Methanobacteriota_WP_056934003.1     LNRFKQI-------------KDLNENVAFVYFGD--------------------------
AA_Firmicutes_NP_389278.2               HDDIKQI-------------KDNDDYVAMAYIGT--------------------------
AA_Desulfobacterota_WP_144684422.1      ITFLKLI-------------EASSPHLVEVFLGS--------------------------
AA_Chloroflexota_WP_116224856.1         IEILNTY-------------RTTHPYVNSVYMGR--------------------------
AA_Halobacterota_WP_004039109.1         IDILRAY-------------QISHPYVSSVYMGR--------------------------
AA_Thermoplasmatota_OPX61375.1          IDVLKDF-------------QDSHEYVNSCYMGR--------------------------
AA_Spirochaetota_WP_018527208.1         YETLRQFGPGHVNHHPFLQSSGYDDAVGTLYLAF--------------------------
AA_Singergistota_WP_012869308.1         ARYFKA--------------SAKELNVLEMYVGLE-------------------------
AA_Verrucomicrobiota_WP_176014353.1     HEMFKFM-------------FANLDSVVLAYLGT--------------------------
AA_Planctomycetota_MBG80894.1           YNFNEQL-------------IDQNDLIAGSSIAFEPG----------AWE----------
AA_Campylobacterota_WP_010891944.1      INNVGPI----------LKYYRHSINALNVYLGL--------------------------
AA_Chrysiogenetota_WP_183731730.1       MERWQTL-------------LDYSNDIFFIYFGD--------------------------
AA_Acidobacteriota_HCZ33114.1           AQLVTPL-------------LAAQRSITSLNLARADGHSLLFLRFGGAWSLRELEAP---
AA_Deinococcota_WP_184108923.1          SVTFQTM-------------LNAVPQLNGVLIGHDDG-----------------RFTSTR
AA_Thermodesulfobacteria_RUM89741.1     NRKFFPL-------------LKNQKQVTGLILADSTGREYFL-----YLDGDSWVTRVSS
AA_Fibrobacterota_WP_022637451.1        EPWFQEIARQA---------REHTPQISTVNYGNIYKNYLGI----RINDDESLNLMVQD
AM_Desulfobacterota_WP_027185430.1      STDLLGF-------------ILFNSAVYGSCVAYEPY----------AFD-RDVEF----
AM_Aproteobac_McpX_WP_014528895.1       INLLKAN-------------LEQHGFAFGSWFAEEPK----------AYDGKDVIDNTER
AM_Gproteobac_PacA_WP_039291840.1       DKMMEYA-------------LRDNPEYLSISVIFEEN----------VFDGRDAEFADQP
AM_Halobacteriota_WP_011034866.1        LLILENL-------------LRDNPHLLGTYVAFEPD----------AFDGKDAEYTNSP
AM_Spirochaetota_WP_015707342.1         NDILKNA-------------LANKEYITAFCIAYDPN----------AMDGKDAQYAGQG
AM_Verrucomicrobiota_WP_129046516.1     DAMLRGS-------------LEGNTDYIGVWTLWEPN----------AFDGRDADYVNKP
AM_Aproteobac_WP_041812266.1            SIWLKSI-------------AEANPDFLGVWVGMEPN----------ALDGRDAEFANKP
AM_Bacteroidota_WP_092437134.1          QQMAGSI-------------LLGDEDFLGYTVCFEPN----------AYDAKDAFFANKP
AM_Firmicutes_WP_056043800.1            VRLIENF-------------VREQPYILGVFTVWEPN----------AFDNQDGNFRNKS
AM_Firmicutes_WP_052635864.1            SEIFKNA-------------LADNPQFIGISIAFEPN----------AFDSLDAQFDGDS
AM_Gproteobac_PctD_WP_038403940.1       SALLRST-------------VQNNPKLLDTFMAWEPN----------AFD-TDAAFAGQP
PU_Gproteobac_R3_WP_124259872.1         KTYAQTL-------------KNESHAATIAWVSE--------------------------
PU_Gproteobac_McpH_WP_047603170.1       IEYLTAA-------------KQRNHAFTTLFAST--------------------------
```

```
PU_Gproteobac_R1_WP_219614703.1         IDQLKDM-------------TAQYGLVTASFADR--------------------------
PU_Firmicutes_A_R5_WP_131005693.1       QDYLNVY-------------RNKYSYDSVFLVST--------------------------
PU_Actinobacteriota_R8_WP_227113344.1   CAYLESY-------------REAFGFDSVFLVST--------------------------
PU_Cyanobacteria_R9_WP_198537540.1      QRYLSRV-------------QAQHGATTVFFVSE--------------------------
PU_Gproteobac_R7_WP_038903150.1         TRYLREI-------------DRRFNTVVSFFVSN--------------------------
PU_Gproteobac_R6_WP_185834821.1         IRYLREI-------------QRQYQTISSFYISD--------------------------
PU_Verrucomicrobiota_R10_WP_110129388.1 VRYLHEI-------------KIKYGTVSSFFVSD--------------------------
PU_Verrucomicrob_R11_WP_162024566.1     TKFLHEI-------------KVEYSTISSFFVSD--------------------------
PU_Verrucomicrobiota_R12_WP_220621668.1 QRYLEEI-------------RREYGTVTSFFISE--------------------------
PU_Campylobacterota_R2_WP_107944080.1   FNYNTDL-------------MKAFNLSTAMFVSD--------------------------
PU_Firmicutes_R13_WP_167859577.1        GRKLTLL-------------TSEHGYDNSFLVSA--------------------------
PU_Firmicutes_R15_WP_207952809.1        LQKLDNL-------------AKGFDYSNGFIASR--------------------------
PU_Firmicutes_R14_WP_021170906.1        KTKITDI-------------GKNYDYVKAFVAST--------------------------
PU_Gproteobac_R4_WP_199478134.1         AEELNSI-------------KQLSGSDSTFYVVNM-------------------------

AA_Proteobacteria_NP_252999.1           ----------------------------QDG-VFTMRP-----------DSPMPAGYDP-
AA_Cyanobacteria_WP_162544314.1         ----------------------------SEG-QFFQWP-----------PSTLPEGYDP-
AA_Actinobacteriota_WP_187050447.1      ----------------------------KDG-KTLIFP-----------KADLPADFDP-
AA_Bacteroidota_WP_196119247.1          ----------------------------EDG-SIFITP----------IDPTMPDDYDC-
AA_Thermotogota_WP_114702178.1          ----------------------------SDK-KFYIYP-----------DAELPEGYDP-
AA_Methanobacteriota_WP_056934003.1     ----------------------------ENG-NMYLWP-----------DEPLPPDYDP-
AA_Firmicutes_NP_389278.2               ----------------------------AKK-EMFTYP-----------KADFAEDYDP-
AA_Desulfobacterota_WP_144684422.1      ----------------------------RWG-GFTYAG-----------EDALPAGYDP-
AA_Chloroflexota_WP_116224856.1         ----------------------------ENG-SFVRSH-----------PRNEPTQYDP-
AA_Halobacterota_WP_004039109.1         ----------------------------ENG-AFVRSY-----------ERARPTAYDP-
AA_Thermoplasmatota_OPX61375.1          ----------------------------ENG-AFVRAY-----------PRASPTQYDP-
AA_Spirochaetota_WP_018527208.1         ----------------------------RDG-VHTTGS-----------AWNIPDDYDP-
AA_Singergistota_WP_012869308.1         ----------------------------STG-KVGTGG-----------DWVEKPDYDA-
AA_Verrucomicrobiota_WP_176014353.1     ----------------------------VDG-VELQYP-----------WSHTPLAYDP-
AA_Planctomycetota_MBG80894.1           ----QETDRYAPYSWI------------TDG----------S-----TNRRDLALDYDY-
AA_Campylobacterota_WP_010891944.1      ----------------------------NNG-KVLLSQKSND-----AKMPELRDDLDI-
AA_Chrysiogenetota_WP_183731730.1       ----------------------------REG-NVTMLP----------DDEPLPPDFNM-
AA_Acidobacteriota_HCZ33114.1           --GPGARIRW--------------RRLDQNG-QVLSNE------------PWTPMAYDP-
AA_Deinococcota_WP_184108923.1          RGGSSDAGRSLSTIETRPTRRATVTVLDERG-RIVSRR-------------VDTGNYDP-
AA_Thermodesulfobacteria_RUM89741.1     V---------------------------KGSVTHMVFQKWNGPDEPVKKWEKNVRDYDP-
AA_Fibrobacterota_WP_022637451.1        TH--------------------------TND-SLYIYETLHRS----GPIAAVFGAYDP-
AM_Desulfobacterota_WP_027185430.1      ---------FAPYAYM------------PGG-RPMFTY--------------LSADYNY-
AM_Aproteobac_McpX_WP_014528895.1       --GGNADGAFTPYWSKD-----------RNG-NIQLST------------------FKAD
AM_Gproteobac_PacA_WP_039291840.1       --GQAPKGRYAWFVDRD-----------QAG-NYAMHP---------------LLSYLTP
AM_Halobacteriota_WP_011034866.1        --AHDGTGRFVPYWNK------------MNG-TASVAP---------------LLHYDS-
AM_Spirochaetota_WP_015707342.1         P-AYDETGRYAPYWNK------------LGG-NIDVEF---------------LPDIDS-
AM_Verrucomicrobiota_WP_129046516.1     --GHDATGRYIAYWNR------------GSG-KVIVEP---------------LVDYTTE
AM_Aproteobac_WP_041812266.1            --GSDASGRFLPYWNR------------GSG-TVALES---------------LVGYDEP
AM_Bacteroidota_WP_092437134.1          --GHDNTGRFVSYMTKN-----------GSG-GFVVEP---------------LVDYENE
AM_Firmicutes_WP_056043800.1            S-YDDDTGRFVPYIVR------------QGD-KIVAYP---------------NKNYENI
AM_Firmicutes_WP_052635864.1            --RYYEKGQFATYFVRGN---------LNSG-KDMYST------------ITQEPLRDL-
AM_Gproteobac_PctD_WP_038403940.1       GKGYGPDGRYLPWWYRG-----------ADG-KPIVEA--------------MADSIDSE
PU_Gproteobac_R3_WP_124259872.1         ----------------------------KTG-KYLDEN-----------GFSRTVQRSD-
PU_Gproteobac_McpH_WP_047603170.1       ----------------------------ETG-HYYNEN-----------GLDRTLSRSN-
PU_Gproteobac_R1_WP_219614703.1         ----------------------------QSA-AYYNQD-----------GFLRNLTP---
PU_Firmicutes_A_R5_WP_131005693.1       ----------------------------KTN-NYYHFN-----------GLDRNLSANN-
PU_Actinobacteriota_R8_WP_227113344.1   ----------------------------ESN-RYYHFN-----------GVDRTLERDN-
PU_Cyanobacteria_R9_WP_198537540.1      ----------------------------ASR-RYYHPT-----------GILKTVSPGS-
PU_Gproteobac_R7_WP_038903150.1         ----------------------------NTH-RYYDPE-----------KISHTLLETS-
PU_Gproteobac_R6_WP_185834821.1         ----------------------------RTG-HYYHYT-----------GILKQVSESN-
PU_Verrucomicrobiota_R10_WP_110129388.1 ----------------------------KTL-KYYYAH-----------GLLKTVSEDE-
PU_Verrucomicrob_R11_WP_162024566.1     ----------------------------ISH-NYYHAQ-----------GLLKEVKENE-
PU_Verrucomicrobiota_R12_WP_220621668.1 ----------------------------KSR-NYYYWG-----------GVLKQVDENE-
PU_Campylobacterota_R2_WP_107944080.1   ----------------------------KTL-NYYTND-----------KILKQLSKDN-
PU_Firmicutes_R13_WP_167859577.1        ----------------------------VSN-RYWGEG----------GTVLRTMSRSD-
PU_Firmicutes_R15_WP_207952809.1        ----------------------------MTG-SYWIEG----------KQMSKILSQTN-
PU_Firmicutes_R14_WP_021170906.1        ----------------------------VTN-QYWEDN-----------KVVKPLSKTS-
PU_Gproteobac_R4_WP_199478134.1         ----------------------------KSGLEFLGYD----------QKFFRTPLADY-

AA_Proteobacteria_NP_252999.1           -------RSRPWYKDAVAA---GGLTLTEPYVDAA-TQE--LIITAATPVKA-A---GNT
AA_Cyanobacteria_WP_162544314.1         -------RKRPWYQAAASF---GKLTLTEPYIAAS-SGE--LNITAAAPRYQ-G---GQL
AA_Actinobacteriota_WP_187050447.1      -------RQRDWYQSALKQ--KNKTIWTEPYTDQA-TNE--LVITAAKAIYDDR---DEL
AA_Bacteroidota_WP_196119247.1          -------RTRSWYKATVNN--NEKIIWTEPYVDAGDLGN--VIVTVAKAIHK-N---DSL
AA_Thermotogota_WP_114702178.1          -------TKRPWYVDAVKN--KGKVIITEPYLDAS-TSD--IVITVAKAVVN-N---GQI
AA_Methanobacteriota_WP_056934003.1     -------RVRPWYIKAKEN---NGPSYTEPYRDAF-TGK--WVITYSEPVYV-D---GKF
AA_Firmicutes_NP_389278.2               -------TSRPWYKLAAET--PDQVVWTEPYKDVV-TGD--MIVTASKAILD-R---QKV
```

```
AA_Desulfobacterota_WP_144684422.1      -------RSRPWYQTALAN--PARTSITSAYMST--TGE--PVISLMHGIQSER---NEL
AA_Chloroflexota_WP_116224856.1         -------RDRPWYILAKEN--PGTVMITEPYQSLT-TSD--INVGIVTALVDQT---GEV
AA_Halobacterota_WP_004039109.1         -------RERPWYILAKEH--PGEVMVTEPYSSVT-TPD--VNIGTVTALLDED---GEV
AA_Thermoplasmatota_OPX61375.1          -------RERPWYQLAMET--PGEVRITDPYSAVT-TDD--VNIGIVLALMD-N---GTV
AA_Spirochaetota_WP_018527208.1         -------RERGWYQTAQSQ---TGPGFSDSYVDAH-TGN--LVITAARPLRDQE---GEI
AA_Singergistota_WP_012869308.1         -------RKRPWYIQAVQE---DKVILTAPYVDAN-TGG--LVITVATPVKSTS---GKL
AA_Verrucomicrobiota_WP_176014353.1     -------RQRGWFKSAAAH--PGETCWSAPYASASLDNK--LVISCSRAIQDAD---GKL
AA_Planctomycetota_MBG80894.1           -------LERDWYRVGVD----GEAGWTEPYDGPV-FGS--LLVTYSSPVIQ-N---GKV
AA_Campylobacterota_WP_010891944.1      -------KTKDWYQEALKT---NDIFVTPAYLDTV-LKQ--YVITYSKAIYK-D---GKI
AA_Chrysiogenetota_WP_183731730.1       -------FERPWYLRATES--PGQIAWTDLYDEII-TGI--PQISAVVTVHDPE--SLEP
AA_Acidobacteriota_HCZ33114.1           -------RTRPWYLAGAAA---PAPTWTEPYAFYT-TQD--PGITYTLPVRD-T---TGL
AA_Deinococcota_WP_184108923.1          -------RTRPWYTLAVAT--PGITVWTPPYTFAS-SGQ--PGVTVARAVDA-G---ADG
AA_Thermodesulfobacteria_RUM89741.1     -------RKRPWFRSSSSE---RRVFWSPVYRFYS-TGK--PGVTASVSWAESG--NPPR
AA_Fibrobacterota_WP_022637451.1        -------TQRPWFAPLFES---PTKQWSEVYVNLDEKAE--LTITHMLPVFSPQ---NTL
AM_Desulfobacterota_WP_027185430.1      -------PQADWFLIPKEI---RRPIWSEPYFDEG-GGN-VVMSTYSIPFFREEDGRKRF
AM_Aproteobac_McpX_WP_014528895.1       -------YAAEWYGLAAKS---GKGAITQPYLAEG-TDVPTTMTSIAYPVMS-N---GRM
AM_Gproteobac_PacA_WP_039291840.1       -------GQGDYYLLPQKS---QKDTLIEPYTYAY-NGVPTLLTSVAAPIVS-Q---GKL
AM_Halobacteriota_WP_011034866.1        ---------SDYYQLPKAT---EKDVLTEPYFYEG-----VFMVSYVSPIMK-E---GEF
AM_Spirochaetota_WP_015707342.1         ---------EDWYIVPKAE---RHEYITDPYPYGL-QGRTVMLASLIFPIIH-S---DKF
AM_Verrucomicrobiota_WP_129046516.1     -------GAGDYYLLAKHS---NQETVLEPYIYKV-AGRDVLMTSLVVPVNRAD---GTF
AM_Aproteobac_WP_041812266.1            G------SDGAYYQIPKRT---GHAMVVEPYSYTV-AGRKVLMVSMSVPIVE-N---GRV
AM_Bacteroidota_WP_092437134.1          -------SAAPWYWIPMRT---MKEFVTEPLMYPI-QGKNVYMVSFMCPIIT-N---GKF
AM_Firmicutes_WP_056043800.1            -------GDGDYYQIPKRT---KKFALMEPYYYDI-NGERILISSFVYPILDEQ---GKF
AM_Firmicutes_WP_052635864.1            -------EISDYYIVPKQT---LSNVMIEPYIYEV-QGKEVLMTTCSSPIVI-D---GKY
AM_Gproteobac_PctD_WP_038403940.1       KLLPTGVRENEFYACPKEN---KRPCIIDPAPYEM-GGKTVMMSSFNVPIMV-G---DQF
PU_Gproteobac_R3_WP_124259872.1         -------ATDNWFYDFLSK--GKQLEIKLGKDKAS--SV--YNLFIDARFDV-N---GK-
PU_Gproteobac_McpH_WP_047603170.1       -------PKDKWFYGYIDS--GAERFINIDIDGAT--GE--LALFIDYRVEK-E---GKL
PU_Gproteobac_R1_WP_219614703.1         -------EQDAWFYGYTKS--PQDLMLSI-FRETN--GE--VKLFVNFQQLN-----GR-
PU_Firmicutes_A_R5_WP_131005693.1       -------SENQWYYTFLKN--DDEYSLNVDNDEAS-NNS--ITVFVNCKIKDDN---GAT
PU_Actinobacteriota_R8_WP_227113344.1   -------PENTWYFDFLDR--DEAYSLNVDNDEAT-ADE--ITMFVNARILDEQ---GST
PU_Cyanobacteria_R9_WP_198537540.1      -------AQDAWFFRLRAS--ASSYEVNLDRDTAD-PSR--TTVFVNYKLLGDG---GRF
PU_Gproteobac_R7_WP_038903150.1         -------PEDKWFFDIRDEKDGDPYDIEIGVDPEN-RTR--MDIFINYKVFDYS---GNF
PU_Gproteobac_R6_WP_185834821.1         -------PNDAWFYRVKNSAPDKNFEVNIDIDTAN-SQQ--TVVFVNYKVFDFE---NRF
PU_Verrucomicrobiota_R10_WP_110129388.1 -------PRDEWYFRVREM--DEPYEINVDPDMAN-QDA--LTIFINYRVRDYA---GNF
PU_Verrucomicrob_R11_WP_162024566.1     -------PRDAWYFKARDM--DAPYEINVDLDMAN-QDQ--MTIFINYRVFDYN---DNF
PU_Verrucomicrobiota_R12_WP_220621668.1 -------PRDVWYFRVREM--EKPFEINVDIDMAN-NDA--LTVFVNYRVYDYE---GNY
PU_Campylobacterota_R2_WP_107944080.1   -------PRDSWYFDVKNG--KEVNSLNIQVSEAT--GS--LTLYVNSKVEK-D---GKF
PU_Firmicutes_R13_WP_167859577.1        -------PEDAWFYANVDN--RKPIEIVLDYAIAR--GD--TFVFVNALIGG----AERA
PU_Firmicutes_R15_WP_207952809.1        -------PADKWFYDSFAS--QKRMALNIDYNDSR--RD--TFVFVNVLVGD----VESP
PU_Firmicutes_R14_WP_021170906.1        -------YTDKWFYKALKS--GKKIELNIDYDSIH--NE--TFIFFNTLVGD----VRQP
PU_Gproteobac_R4_WP_199478134.1         -------PYKEFYPNFLAK--NKDYELNLQYADQK--------LYINYRSREMDPTTGKP

AA_Proteobacteria_NP_252999.1           LGVVGGDLSLKTLVQIINSLDF--S-GMGYAFLVSGDGKILVHP-DK----------EQV
AA_Cyanobacteria_WP_162544314.1         VGVVGSDFSIQMLDQMLKDANL--G-DLGFLFITDKSGKVLIHA-NQ----------DLI
AA_Actinobacteriota_WP_187050447.1      IGVLGVDISIDTLITMVNQTKF--G-ETGYTVLLDQKGSFVTHP-DK----------EKI
AA_Bacteroidota_WP_196119247.1          VGVIGMDIKLKKFSNIINNIQF--G-ENGYLMLLSKKGDVYAHP-NN----------KML
AA_Thermotogota_WP_114702178.1          IGVVALDFKASELANSLLSNKF--G-ENGYSYLLSSDGKTLLHV-DE----------EKI
AA_Methanobacteriota_WP_056934003.1     VGVVGIDVFVSTLMKEAMDIKL--G-KSGYIVLVNQEGLVLMHP-KE----------EYI
AA_Firmicutes_NP_389278.2               IGVASYDLKLSAIQSMVNKQKV--P-YKGFAFLADASGNLLAHP-SN----------Q--
AA_Desulfobacterota_WP_144684422.1      VGIAGLDVSLGALTSLIQRSQI--G-ETGYVMLVQGDGVILANPRNP----------ETN
AA_Chloroflexota_WP_116224856.1         YGVIGADVTLTNLTEFISGFDV--G-HSGQLLLVNEQGAILANK-NS----------QLL
AA_Halobacterota_WP_004039109.1         YGVIGADITLVNLTEYLASIES--I-GNQEMILTDRSGTILASY-NT----------TVL
AA_Thermoplasmatota_OPX61375.1          YGVVGADITLVGLTDYLRQASE--V-SGRDMLLSDDRGTILAYK-QE----------QLL
AA_Spirochaetota_WP_018527208.1         FGVLATDISLGQITEAISEYCL--G-EGGYAFLVNQEGSLLAHP-DT----------EXA
AA_Singergistota_WP_012869308.1         LGVAGIDVDLKTLSDMITSYKV--F-GKGYGFLLDREGNMICHP-KS----------EMI
AA_Verrucomicrobiota_WP_176014353.1     FGVLALDISVEAVIHDFISTQE--M-RRGSAFLLNDSGEIIAQE-GM----------DLL
AA_Planctomycetota_MBG80894.1           IGVMAADIALLPLQQRLKEEAP----EGLSTILASASGRLIAHP-NP----------ALI
AA_Campylobacterota_WP_010891944.1      IGVLGVDIPSEDLQNLVAK-------TPGNTFLFDQKNKIFAAT-NK----------ELL
AA_Chrysiogenetota_WP_183731730.1       VGVMALDVSLHQLRRIVMDVEL--P-SDAELVLLDRNHQLIVSSTGK-------------
AA_Acidobacteriota_HCZ33114.1           RGVAALDFLLDDLTAQVWTAQP--T-SRSRCLVVDAQGRALVLPRDPAFETPEGRRRAFL
AA_Deinococcota_WP_184108923.1          TVVVGADVQLRQVASFLQGVQI--G-GHGRAFVTDAQGHVIATS-PT---WPG----NVT
AA_Thermodesulfobacteria_RUM89741.1     TVVFALDITLSQIQKFLELQDD--N-KHELLFILNPRDKFFITP-------------KYI
AA_Fibrobacterota_WP_022637451.1        YGAAIVDVKLGRLHAFLQEEEI--P-PGGAVYITTSEGSLLASSYKT---------QDTI
AM_Desulfobacterota_WP_027185430.1      LGVVTADISLEWLRTFIKSISI--Y-QSGYAFLLSRNGVFLSHP-ND----------QFI
AM_Aproteobac_McpX_WP_014528895.1       IGVSGVDISLAALADRLSAVKP--F-GSGRVYLLSQSGKWLAAP-IP----------ELL
AM_Gproteobac_PacA_WP_039291840.1       WGVVTSDISLASLQQKINQIKP--WEGGGYAMLLSSAGKVISYP-DK----------SQT
AM_Halobacteriota_WP_011034866.1        AGIGGVDVSLEYVDEVVSKVRT--F-DTGYAFMVSNSGVILSHPTHK----------DWI
AM_Spirochaetota_WP_015707342.1         IGIISSDIVLDKLQEMVDKVNPH-G-QEGYTEIISHSGAVIAHP-NK----------DYL
AM_Verrucomicrobiota_WP_129046516.1     AGVVGVDLPLETLGAEIAKVKV--G-ETGYAALVSNTGIYAAHP-RA----------ERL
AM_Aproteobac_WP_041812266.1            IGVAGIDLSTDGIWSMLKTVKP--F-DSGSIHLISNDGVWAGHP-DS----------ERM
AM_Bacteroidota_WP_092437134.1          VGVTGVDLSINYLQDMVVKANV--FDGHGNFDIVSHQGVFAANSGNP----------DFV
```

```
AM_Firmicutes_WP_056043800.1          LGVVGADISLDMVQQEVEKIRP----MGGYATMITAGDSYLANGFDR----------ALV
AM_Firmicutes_WP_052635864.1          YGVVGVDIEVDFIKELVKGSEND-M-EFKDILIISSKGNIVGS--KY----------EMA
AM_Gproteobac_PctD_WP_038403940.1     RGAVGADLSLAFIQDLLKRADQQLYDGAGEMALIASNGRLVAYTRDD----------SKL
PU_Gproteobac_R3_WP_124259872.1       IGVAALGLSVNELADFIRKQKI--G-NSGFVYLVSPDGAFVIHR-DA----------ALA
PU_Gproteobac_McpH_WP_047603170.1     VGVAGMGLRMTELSKLIHDFSF--G-EHGKVFLVRNDGLIQVHP-DA----------AFS
PU_Gproteobac_R1_WP_219614703.1       -GLAGLAKSLDSMVSMLANFRI--G-DSGFVFMTDGSGKVKLHP-DA----------ARI
PU_Firmicutes_A_R5_WP_131005693.1     MGIIGVGLKVNSLQMLLKGYND--K-FDVVARLIDDKGFVQLAV-DK----------TGH
PU_Actinobacteriota_R8_WP_227113344.1 LAIVGVGFRMDDLKELLAGFEA--R-TDTRVRLLADDGTIRAST-DP----------NEN
PU_Cyanobacteria_R9_WP_198537540.1    LGAVGLGRSTSQLTRRIQQAER--T-NGIQVMFLDGRGRILFSP-RR----------GQA
PU_Gproteobac_R7_WP_038903150.1       IGVTGVGLPVQRVTQLIETYEQ--R-YNRTIYLIDEDGDVMLHS-KA----------FHR
PU_Gproteobac_R6_WP_185834821.1       LGVIGVGLSSDAVSALVEKYQK--R-YNRHIYFINELGEVTLHG-SH-------------
PU_Verrucomicrobiota_R10_WP_110129388.1 IGATGVGLTVTKVNRLISRYEA--K-YDRQIYFVDASGNVVLRP-SN----------STM
PU_Verrucomicrob_R11_WP_162024566.1   IGMTGVGLTVKNVNNLISHYEA--K-YQRQIYFLNKDGEIVLRP-SN----------SPL
PU_Verrucomicrobiota_R12_WP_220621668.1 IGAAGTGLTVNRVNALIEEYEG--R-FNREIFFVDREGNIILGP-SK----------GRL
PU_Campylobacterota_R2_WP_107944080.1 YGVSAIGMNLDDIVNLVTSKTM--G-EGSKFLMVDSSGIVKIEK------------SDRV
PU_Firmicutes_R13_WP_167859577.1      LGETGVGLSLKQSAEQFQQFKY--G-DKSHLWLVDREGTIYLSD-RY----------EQA
PU_Firmicutes_R15_WP_207952809.1      VGVAGIGLSLKELANNFTNYKY--G-ANSNVWLVDKSGKIYLSD-RV----------EDI
PU_Firmicutes_R14_WP_021170906.1      VAVAGVALSLGDIAKEFGSYKF--G-EHSNLWLVDKQGKIHLAD-DL----------EYN
PU_Gproteobac_R4_WP_199478134.1       LVVAGLAIKVDKLIDMVKQLTI--G-KSGRAMLVTDQGVIQAKG-ESP-------AIDLI

AA_Proteobacteria_NP_252999.1         MK---------TLSEVYPQNT--------------PKIATG------FSEAELHGHTRIL
AA_Cyanobacteria_WP_162544314.1       GK---------TLTDIYPGAQ----------------VSTG-A----MQDVESADGARLL
AA_Actinobacteriota_WP_187050447.1    QQ---------DISKENIFKK--------------MKGESG------SMIEEFEGKSRIV
AA_Bacteroidota_WP_196119247.1        TK---------NISNKEWVKT-------------ILSNEKG------TDIHTWNNEKVVI
AA_Thermotogota_WP_114702178.1        GK---------SVADMDWFKQ-----------MINSKNKSG------VIEYVYDGIKRMA
AA_Methanobacteriota_WP_056934003.1   NK--------LNIYKVPELQE------LA--AELKKGKQEG------TVIYTFEGIRRIA
AA_Firmicutes_NP_389278.2             GK---------NISKDQTLQT-------------IASEKKG-I---------QDVNGKMV
AA_Desulfobacterota_WP_144684422.1    FK---------KLTESGIEAF-----------RVLDKTDRG------SVSLEIKGKEWLA
AA_Chloroflexota_WP_116224856.1       FK---------NVQTMLGESS-----------VEFMKEDHG----------IFTYGRNYC
AA_Halobacterota_WP_004039109.1       FE---------DIGTVLGEQA-----------GFFLATEEG----------VLVHDGRYL
AA_Thermoplasmatota_OPX61375.1        FT---------NVSAVLGDDT-----------DDFLGTEQG----------RIRLENDFF
AA_Spirochaetota_WP_018527208.1       GT---------SLEEIDGYAEV---------ARGILGKGSG-ESGGRQFSTTIAGRPHII
AA_Singergistota_WP_012869308.1       MK--------ENITKPSGLIT-PELAEAG---RKMISGSPG------FVDYSFQGELRRT
AA_Verrucomicrobiota_WP_176014353.1   GQGWN---LQVDLVNLLHSEE-TQLQQLA---AEMIALNSG------CLRLPLGDTVYYV
AA_Planctomycetota_MBG80894.1         LS--------DSLQSEADRTLTPELHAWV---TDIQNQQAG----FQALTSYPDDRAHWL
AA_Campylobacterota_WP_010891944.1    NP---------SIDHSPVLNA------------YKLNGDNN------FFSYKLNNEERLG
AA_Chrysiogenetota_WP_183731730.1     --------NYSQLSDTWQKKT-------------PDQRDRG-------YFSRDNSDMVFA
AA_Acidobacteriota_HCZ33114.1         KQ---------KGTDLLGSQA----------SRMEDDAAQG------LPRRMTSGGQTVI
AA_Deinococcota_WP_184108923.1        GR-------VPSLSEVADPAL----RALI---GPDGQVRPG------TREFTVDRQAYSA
AA_Thermodesulfobacteria_RUM89741.1   GMIIT---AAPSVSETEAAED-SVLIS----RIFQAWEKAG-------------------
AA_Fibrobacterota_WP_022637451.1      TRSEN---GLVSLPKAMQSRH-PLLRQSATALAQKTQDKKN------SLALLIENKEIFV
AM_Desulfobacterota_WP_027185430.1    MR--------ESIFSLAETHSSKVLRDIG---KKMVQGETG----FVRLPEFVMGEPAWL
AM_Aproteobac_McpX_WP_014528895.1     MKEYDG---------------------------------EG-A-----------------
AM_Gproteobac_PacA_WP_039291840.1     SKAWQ---------------------------GPTDNFTSS----VVQHDDAILGEQALV
AM_Halobacteriota_WP_011034866.1      GK--------KDLYDFGGEEL----------EKASRDIKNG-IGGHLETADPTTGKTVIL
AM_Spirochaetota_WP_015707342.1       GK---------DLEETLVEGQ-SRLQHID---EIKSAINSG-------EMYISTGKNFYT
AM_Verrucomicrobiota_WP_129046516.1   GK---------PMKDTDPWVV-----------PFLGNLKKG-EAFETESFSRTLNDMTYR
AM_Aproteobac_WP_041812266.1          GQ---------PIGKSDPALD-----------AAKPAIRAG---RSFEQMSVADGQPVKQ
AM_Bacteroidota_WP_092437134.1        GK--------NILEQKNIGAE-----------DQLVDIEKG-------NSLTRIDNGILK
AM_Firmicutes_WP_056043800.1          SKPYLPLPKGESLEELKEQAL-----------------------TIMYTSDPMLGGTVMR
AM_Firmicutes_WP_052635864.1          YN------QDENLKE------------------EILHFKQG--------SHVEYSNGIFD
AM_Gproteobac_PctD_WP_038403940.1     GE----------------------------------------------------------
PU_Gproteobac_R3_WP_124259872.1       DG-------QHFLRDTSGFNQ-----------EMVTKLLMG--QRFSSVSYSATDGERIA
PU_Gproteobac_McpH_WP_047603170.1     GK--------RQLAEQLGADA-------------AKGVMTG-GESLRSSRFSRDGERYLA
PU_Gproteobac_R1_WP_219614703.1       DR--------DNLTQLASGTT----------ANLLTKQAFA------ATQAEVDGQAVIL
PU_Firmicutes_A_R5_WP_131005693.1     EN--------INFFENSSSDL-----------SNSKQLILNNKKEQKSFWYSSEKSKSYI
PU_Actinobacteriota_R8_WP_227113344.1 GH---------ALFDTEEAAV-------------LSEQTRSDRTDVQDFWYRANGENGFL
PU_Cyanobacteria_R9_WP_198537540.1    PP---------QLQRAIAQQR---------LRDSLREQPRG------AFQFRQGGELIYV
PU_Gproteobac_R7_WP_038903150.1       AS---------NIHQQPGLQS------------LATQVLTS---PGGSYRYSLNGENIFL
PU_Gproteobac_R6_WP_185834821.1       ------HPGFDQIQQREGLKT------------LATQILTS---PSVGASYYADGQKVYL
PU_Verrucomicrobiota_R10_WP_110129388.1 RG-------YDSLQEIEGLGE------------RVADLLAG---KTDSLTYKRLGDRRIM
PU_Verrucomicrob_R11_WP_162024566.1   MN-------YQSLQNIPGLND------------VSTDLLAQ---ERTTLTYEREGTTYML
PU_Verrucomicrobiota_R12_WP_220621668.1 TT-------YGNLDAVPGLKK------------DAQSLTSG--TEEQKIRYERDGKTYFL
PU_Campylobacterota_R2_WP_107944080.1 GK--------VNVKDVLGKEK----------FDVLMNKNGG------VIRHFNGTRNLII
PU_Firmicutes_R13_WP_167859577.1      GT---------KLNTILPGAV---SDELF----------AGFANTQVVTYRTAQTGTIDL
PU_Firmicutes_R15_WP_207952809.1      GA---------MISSFVPEEA---FEQML---GQLNETDSGLAKPVVLDYEDQEGKQMDL
PU_Firmicutes_R14_WP_021170906.1      GR---------LAGDFLPAEV---MQQIL---GDM---DNATARPKVLEYQDSQGRIMDL
PU_Gproteobac_R4_WP_199478134.1       KQ--------TDIASLLQDKN-----------------------QVQIVEKSISGKDYYL

AA_Proteobacteria_NP_252999.1         AFTPI-KGLPSVTWYLALSIDKDKAYAMLSKFRVSA------------------
AA_Cyanobacteria_WP_162544314.1       GFQPI-AGLPGVDWYVAMSIDKGVAFAELSDFRRSTL-----------------
```

```
AA_Actinobacteriota_WP_187050447.1      GY----ATNPTTGWRIAGVMEENEVKVRASPM----------------------
AA_Bacteroidota_WP_196119247.1          SY----LTVPNTQWKLVGVNPIN-IDKEVSPIKNRF------------------
AA_Thermotogota_WP_114702178.1          GFSKM-----DNGWIFVTVALSKEVNKRATE-----------------------
AA_Methanobacteriota_WP_056934003.1     GY----KRLSTTGWIVVATVP---------------------------------
AA_Firmicutes_NP_389278.2               VY----QTIGETGWKVGTQFDTDQLMWISDKMNRA-------------------
AA_Desulfobacterota_WP_144684422.1      EV----HTLPDLGWKLVGFIERDEVMEGFRAMMRL-------------------
AA_Chloroflexota_WP_116224856.1         IY----YTSPQLGWKIAAIIPI--------------------------------
AA_Halobacterota_WP_004039109.1         IY----ITSPELGWKIGTFIPVSTIEERINESIRQTL-----------------
AA_Thermoplasmatota_OPX61375.1          VY----YTSTDMGWKIGTFIPNSYLASEIQES----------------------
AA_Spirochaetota_WP_018527208.1         RT----VTLENFGWHLAMAVP---------------------------------
AA_Singergistota_WP_012869308.1         FFSPT-----RSGFVLGVVFPASDLNAMVRSLAIRQ------------------
AA_Verrucomicrobiota_WP_176014353.1     GY----APVASTDWSLAVVLPEADILKQSHCIEETIATESNRLEEEFQLSIHHQ
AA_Planctomycetota_MBG80894.1           LY----EPVQEANWSMGTAIAEDDILAPVHQQ----------------------
AA_Campylobacterota_WP_010891944.1      ACTKV------FAYTACITESADIINKPIYKA----------------------
AA_Chrysiogenetota_WP_183731730.1       SS----TRIPSTQWQLVLFTPRDSFYAPIDPLRN--------------------
AA_Acidobacteriota_HCZ33114.1           GLSRTFSGLPGLHWKLLLSVPE--------------------------------
AA_Deinococcota_WP_184108923.1          VVQPI-ALAPGVQWRVGVYAPVDDFMLGLQRT----------------------
AA_Thermodesulfobacteria_RUM89741.1     ---------MPLDSFVKVSMDSSKWLASLRPLEQGDEA--------FYVG----
AA_Fibrobacterota_WP_022637451.1        NSTP--FHKDNLHWNIVVAMPEAALMGDVRQRQRNSR-----------------
AM_Desulfobacterota_WP_027185430.1      SY----APVSNSDWSMGLVIPEAEMFQGLEGLSRE-------------------
AM_Aproteobac_McpX_WP_014528895.1       ------------------------------------------------------
AM_Gproteobac_PacA_WP_039291840.1       TWQPVTIGNSTEKWYLGI------------------------------------
AM_Halobacteriota_WP_011034866.1        FYEPV----ETGDFAFVLVVPKEEMLAGVADLRER-------------------
AM_Spirochaetota_WP_015707342.1         VYMPIQFSSVTNPWSVAVSIPMAKILANADSIRNY-------------------
AM_Verrucomicrobiota_WP_129046516.1     FGVPVRIGSSSTPWCVSITIRESEVLAGAWKLRNT-------------------
AM_Aproteobac_WP_041812266.1            LFLPVTVAGTETPWSLLVNLPLDKINAPVRELRNAT------------------
AM_Bacteroidota_WP_092437134.1          AFVPVIVGRCPTAWQVSISVPVDYITQEARAQMIYQ------------------
AM_Firmicutes_WP_056043800.1            LLNP--IHIKDQTWYFETIIPKGNMLKDYYKGLSNT------------------
AM_Firmicutes_WP_052635864.1            VYELINIRDIDDKWGIKLSVDQKTIMGSASRLLANQ------------------
AM_Gproteobac_PctD_WP_038403940.1       --------------------PAGSVL----------D-----------------
PU_Gproteobac_R3_WP_124259872.1         AA----SYVPELNLYIVAEVPQAEILGKITQT----------------------
PU_Gproteobac_McpH_WP_047603170.1       LG----LPLRDLNWTLVAEVPESEIYAQMHQA----------------------
PU_Gproteobac_R1_WP_219614703.1         AT----SYIPMLDWYLVAQVPEAEIYAELDKARLH-------------------
PU_Firmicutes_A_R5_WP_131005693.1       VC----QYIPSLKWHLVLENDFTLMIKQLHLQ----------------------
PU_Actinobacteriota_R8_WP_227113344.1   VS----RYLPNLDWFLVVDHDTSQLDAQMARQ----------------------
PU_Cyanobacteria_R9_WP_198537540.1      RT----KRIPELNWTLVVSQPLRVPSGPLWS-----------------------
PU_Gproteobac_R7_WP_038903150.1         NT----RVIPEFGWKLMVEQNSG-------------------------------
PU_Gproteobac_R6_WP_185834821.1         NS----RWVDEFQWYIIVEQKDE-----------------------F-------
PU_Verrucomicrobiota_R10_WP_110129388.1 NC----RYVPELDWYLIVEQSEATMMAPLRQE----------------------
PU_Verrucomicrob_R11_WP_162024566.1     NC----RFIPELNWFLIIEQSEQELLAPIKEQ----------------------
PU_Verrucomicrobiota_R12_WP_220621668.1 NS----RWIPELNWYLMVEQSEDELLSPLRHTLL--------------------
PU_Campylobacterota_R2_WP_107944080.1   GS----KYIPSLDWYLFGEMDEDVLLKDLHT-----------------------
PU_Firmicutes_R13_WP_167859577.1        IS----RPIESADLDIVFAIPRSESVPFLHKIRTNT------------------
PU_Firmicutes_R15_WP_207952809.1        IS----YPLKSTDWKLLFQMRRSESVAFLDTIKLN-------------------
PU_Firmicutes_R14_WP_021170906.1        AY----QSTATTDWKLVFQIPRSESIAILSSVK---------------------
PU_Gproteobac_R4_WP_199478134.1         GA----LWVPMLDRFIVIEVPSEQILSPIYQQ----------------------
```

Supplementary Figure 4. Phyletic distribution of purine binding dCache_1 domains. Blue dots indicate domains found in the RefSeq database; orange dots – domains found when the search was extended to include protein sequences from the NCBI non-redundant database. The updated taxonomy is used [2].

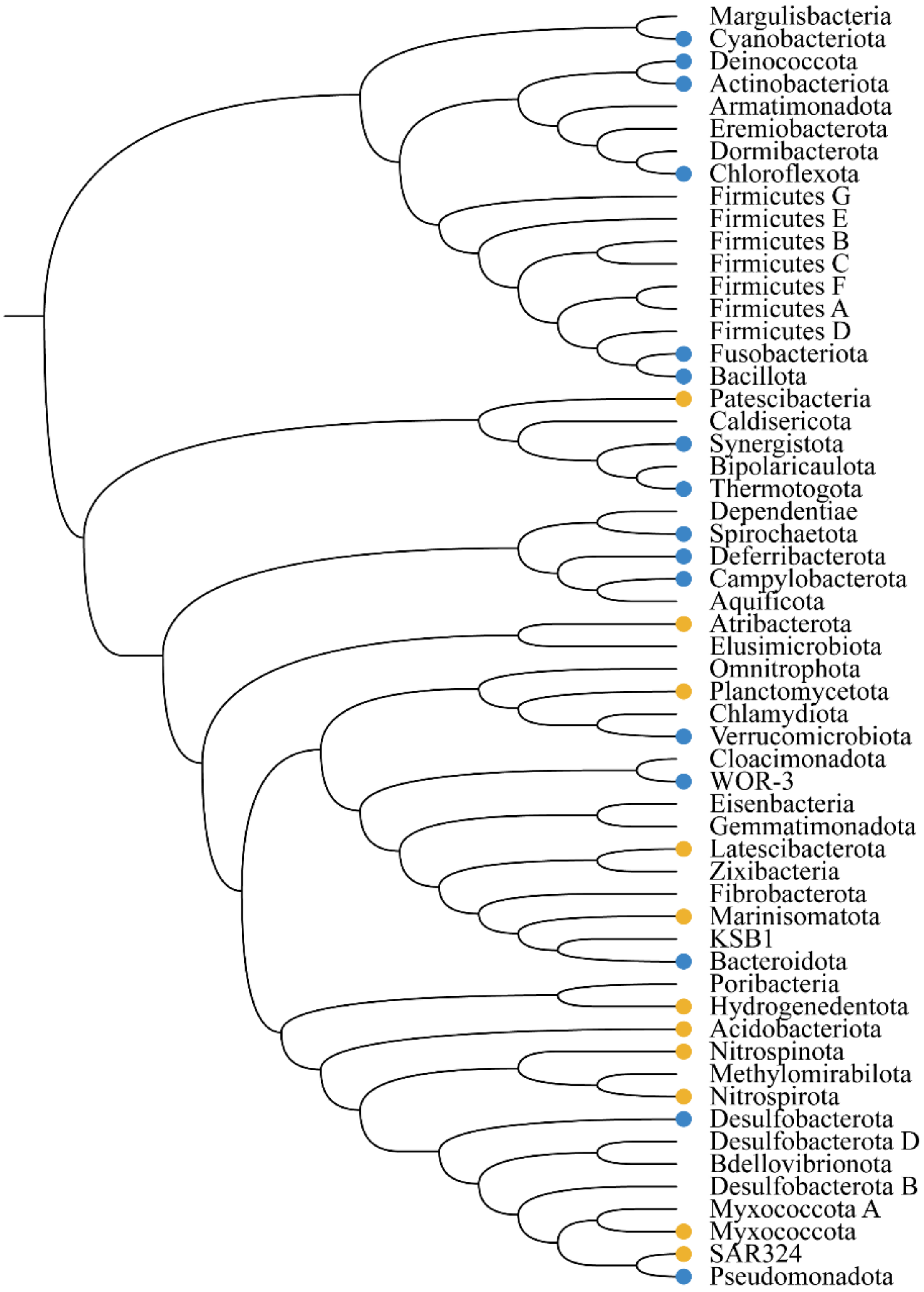

Supplementary Figure 5. Microcalorimetric titrations of the McpH-LBD mutants that were used to derive the dissociation constants provided in Table 1. Proteins at 9 to 18 μM were placed into the sample cell and titrated with 12.8 μl aliquots of 2 mM adenine made up in dialysis buffer. Upper panels: raw titration data. Lower panels: integrated, concentration-normalized and dilution heat-corrected peak areas and best fit using the "one–binding site model" of the MicroCal version of ORIGIN.

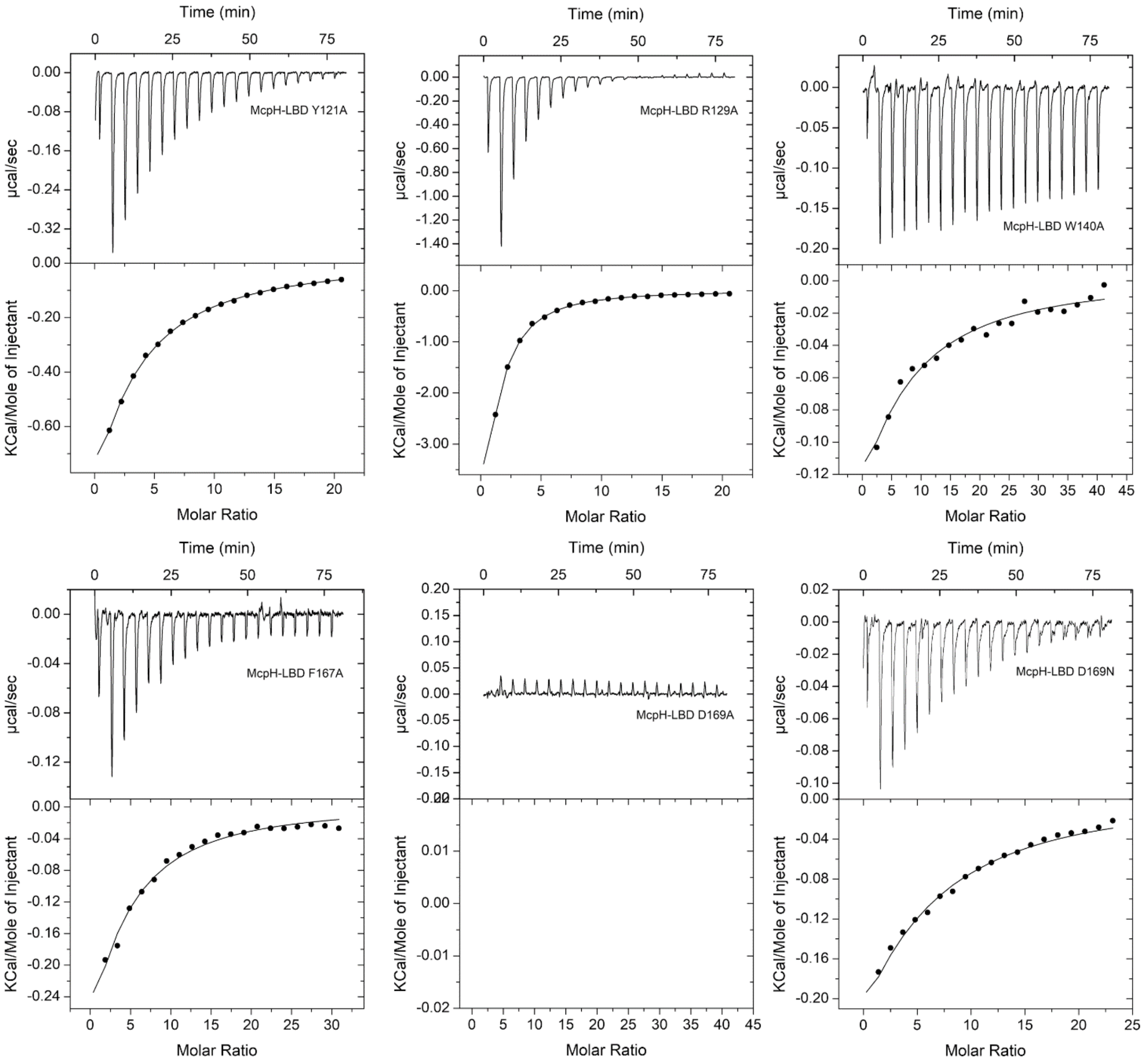

Supplementary Fig. 6. Molecular docking of xanthine (A), adenine (B) and purine (C) to the 3D structure of McpH-LBD. In contrast to uric acid, these three compounds lack a carbonyl group at position C8 but were found to bind with high affinity to McpH-LBD [3]. R129, that in the X-ray structure establishes a hydrogen bond with the carbonyl group at position 8 of uric acid, is shown in stick mode. The corresponding models are available at https://github.com/ToshkaDev/Purine-pyrimidine_motif.

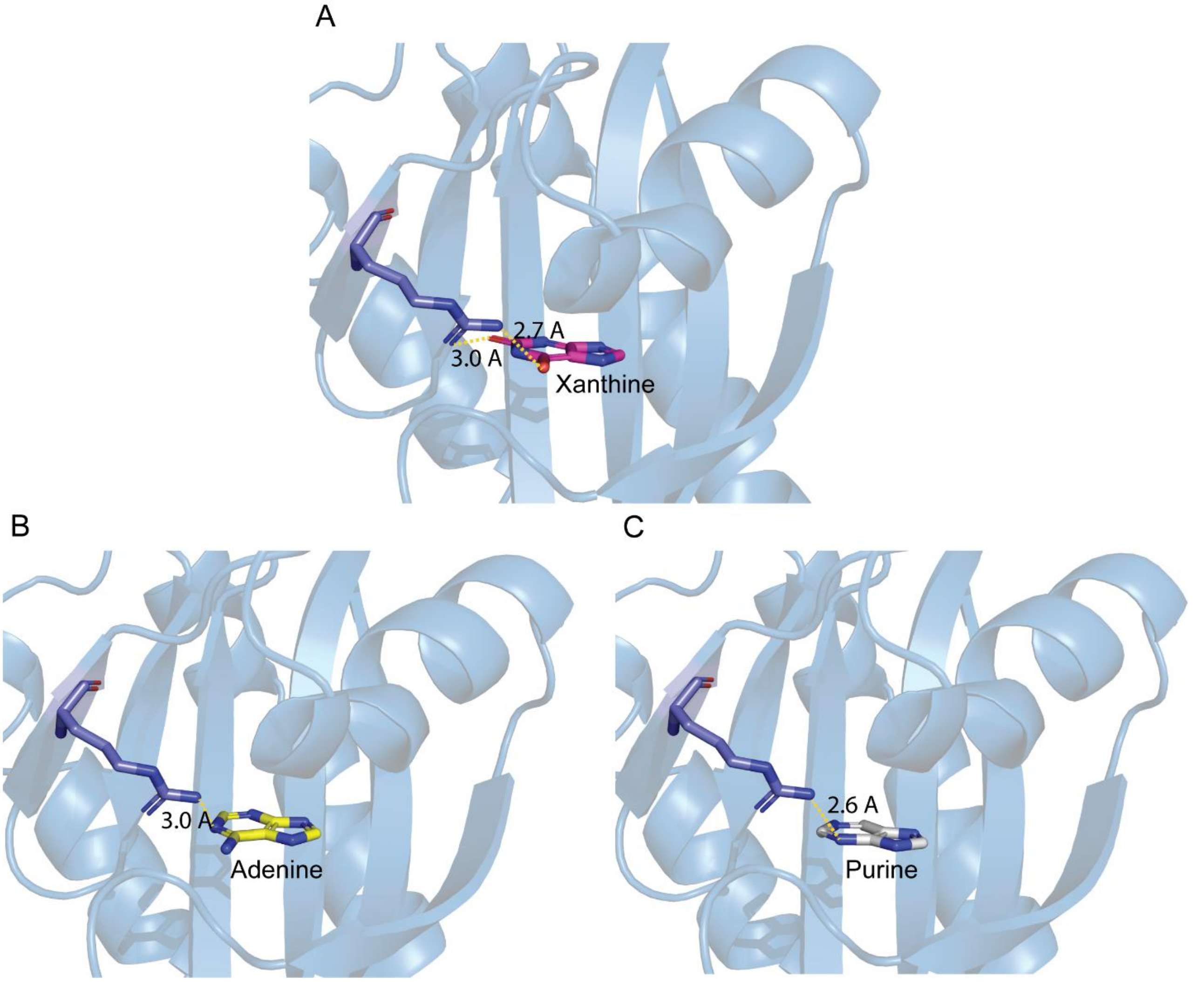

Supplementary Figure 7. Thermal shift assays of R10 and R13. Changes in the midpoint of protein unfolding (Tm) in the presence of 2 mM ligand as compared to the buffer control. The following compounds were used (from left to right): adenine, adenosine, guanine, guanosine, inosine, xanthine, uric acid, allantoin, hypoxanthine, thymine, cytosine, purine, caffeine, theobromine and theophylline.

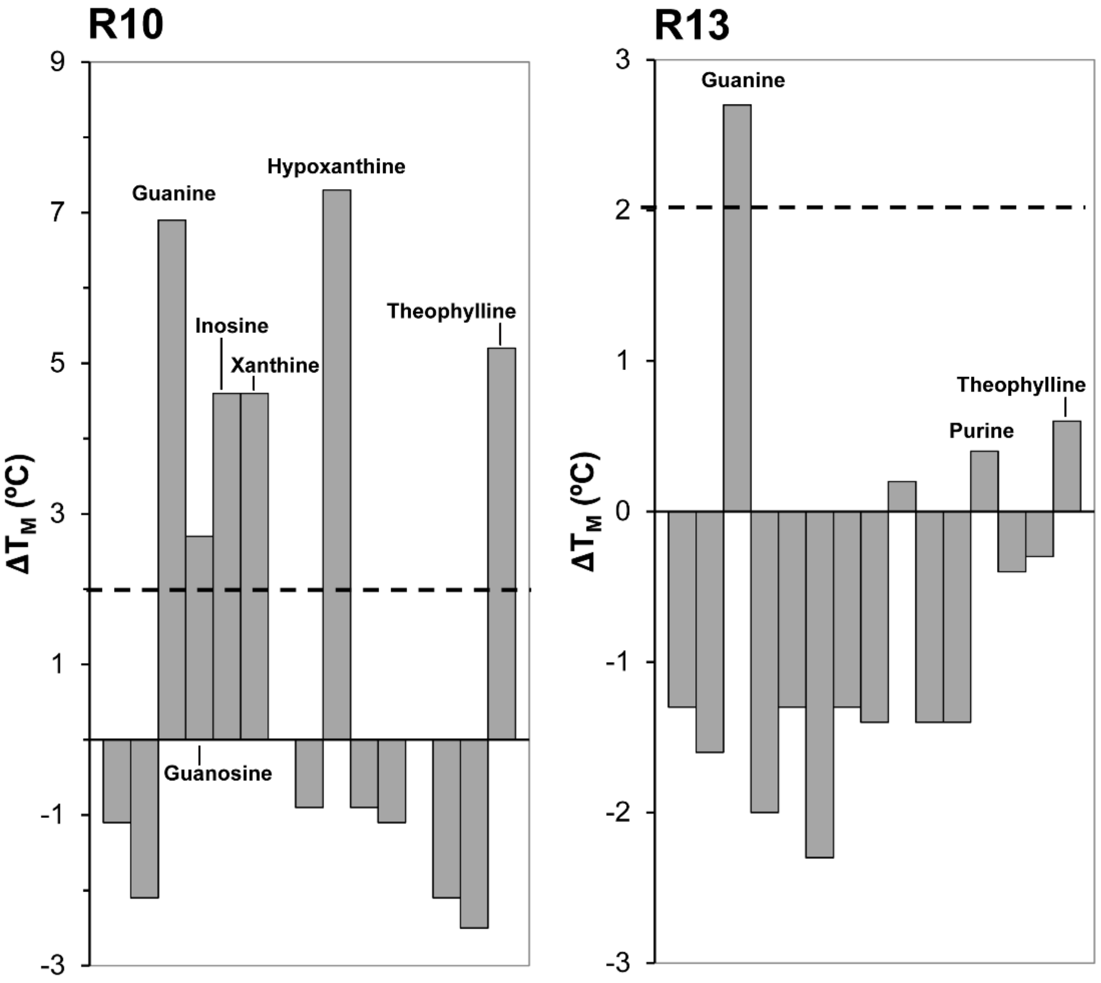

Supplementary Fig. 8. Recognition of purine and pyrimidine compounds by receptor R4. Molecular docking of theophylline (magenta), cytosine (orange) and thymine (grey) to an Alphafold2 model of R4. Shown is a superimposition of the three ligands. The model is available at https://github.com/ToshkaDev/Purine-pyrimidine_motif.

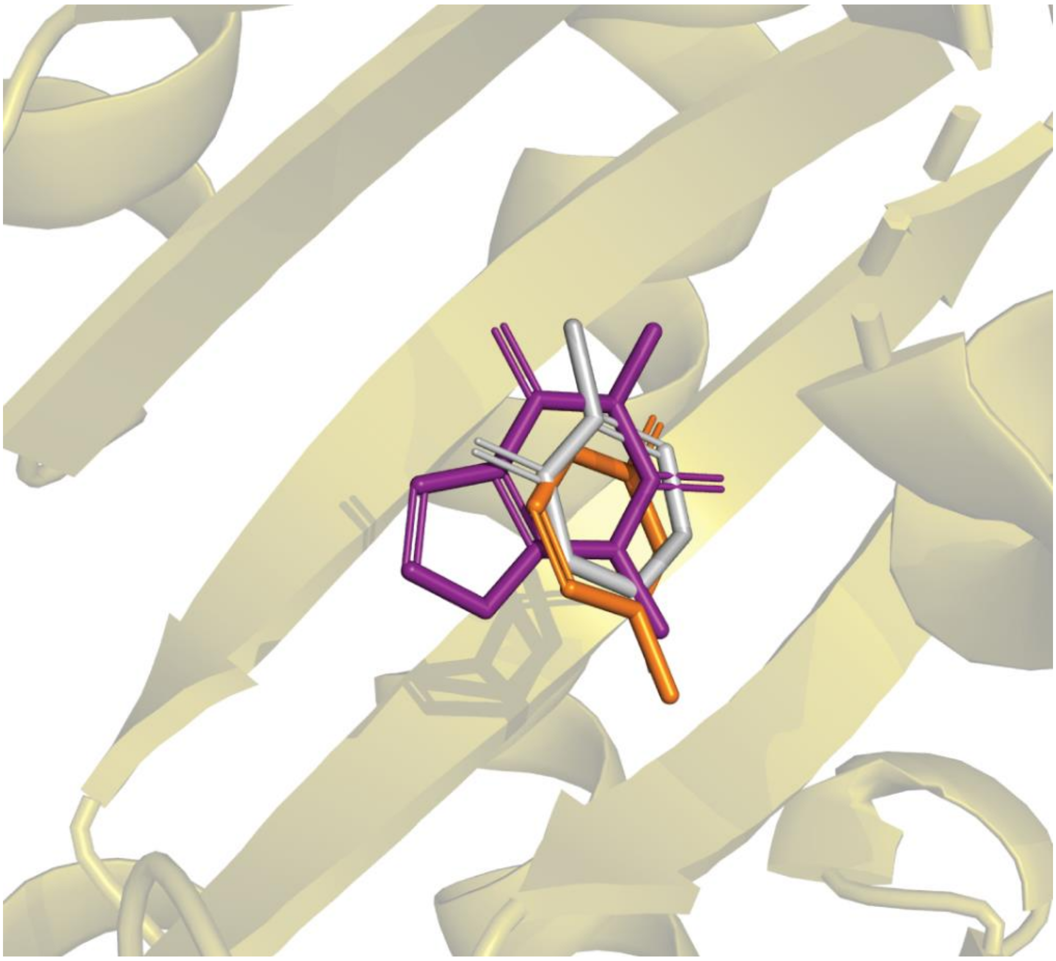

Supplementary Fig. 9. Structure of ligands analyzed in this study.

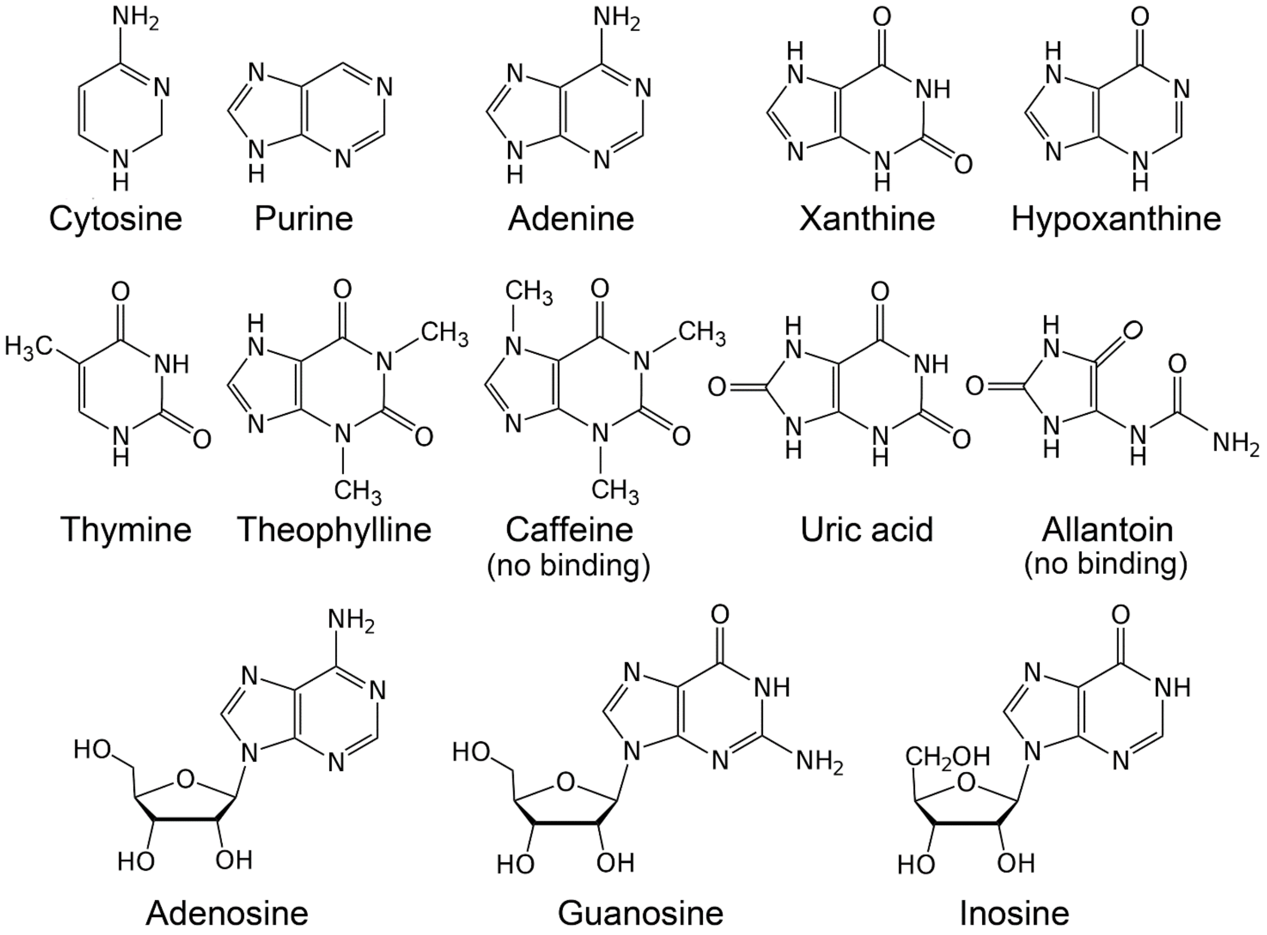

Supplementary Fig. 10. Identification of potential features that may account for the different ligand profiles of domains R11 and R14 that possess the same variant of the purine binding motif (Table 2) but that differ in the ligand profile. Superimposition of the McpH-LBD structure (magenta) with AlphaFold2 models of R11 (pink) and R14 (yellow). Uric acid (present in the McpH-LBD structure) and amino acids at a distance of less than 4 Å from the bound ligand are shown in stick mode. R11 and R14 residues are labelled with the corresponding colour. Models are available at https://github.com/ToshkaDev/Purine-pyrimidine_motif.

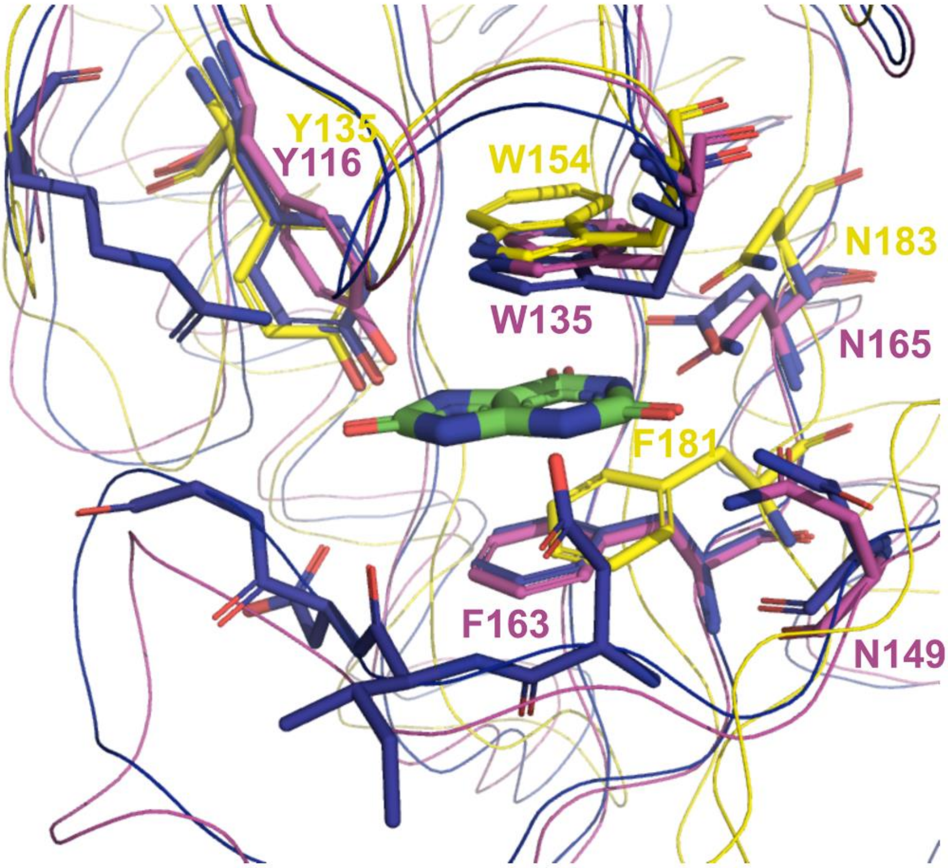

Supplementary Fig. 11. Assessment of the ligand specificity of members of the dCache_1PU family. Microcalorimetric titrations of **I)** 60 µM R4 with 1 mM solutions of theophylline and 15 compounds that were frequent ligands of the amino acid and amine specific families dCache_1AA and dCache_1AM, respectively [4,5], namely: 1, L-Val; 2, L-Pro; 3, L-Trp; 4, L-Ser; 5, L-Gln; 6, L-Glu; 7, L-Asp; 8, L-Arg; 9, acetylcholine; 10, choline; 11, trimethylamine N-oxide; 12, methylamine; 13, dimethylamine; 14, trimethylamine; 15, ethanolamine. **II)** 30 µM R8 with 250 µM solutions of hypoxanthine and the above 15 compounds. **III)** 70 µM R14 with 500 µM solutions of hypoxanthine and the above 15 compounds. Panels A to C are raw titration data. Panel D show the integrated, concentration normalized and dilution heat corrected peak areas of the titration with the purine compound. Data were fitted with the "One binding site" model of the MicroCal version of ORIGIN.

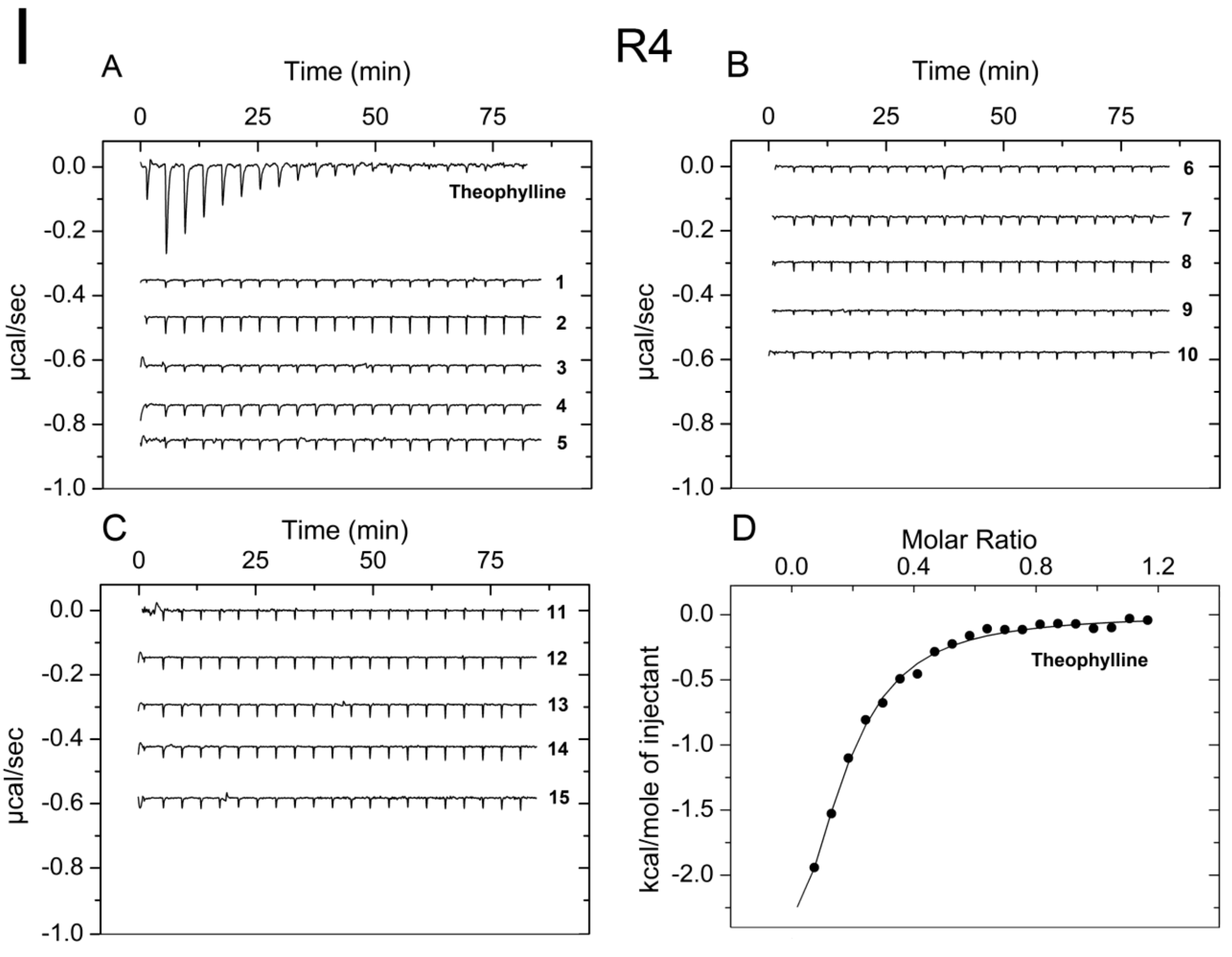

II

R8

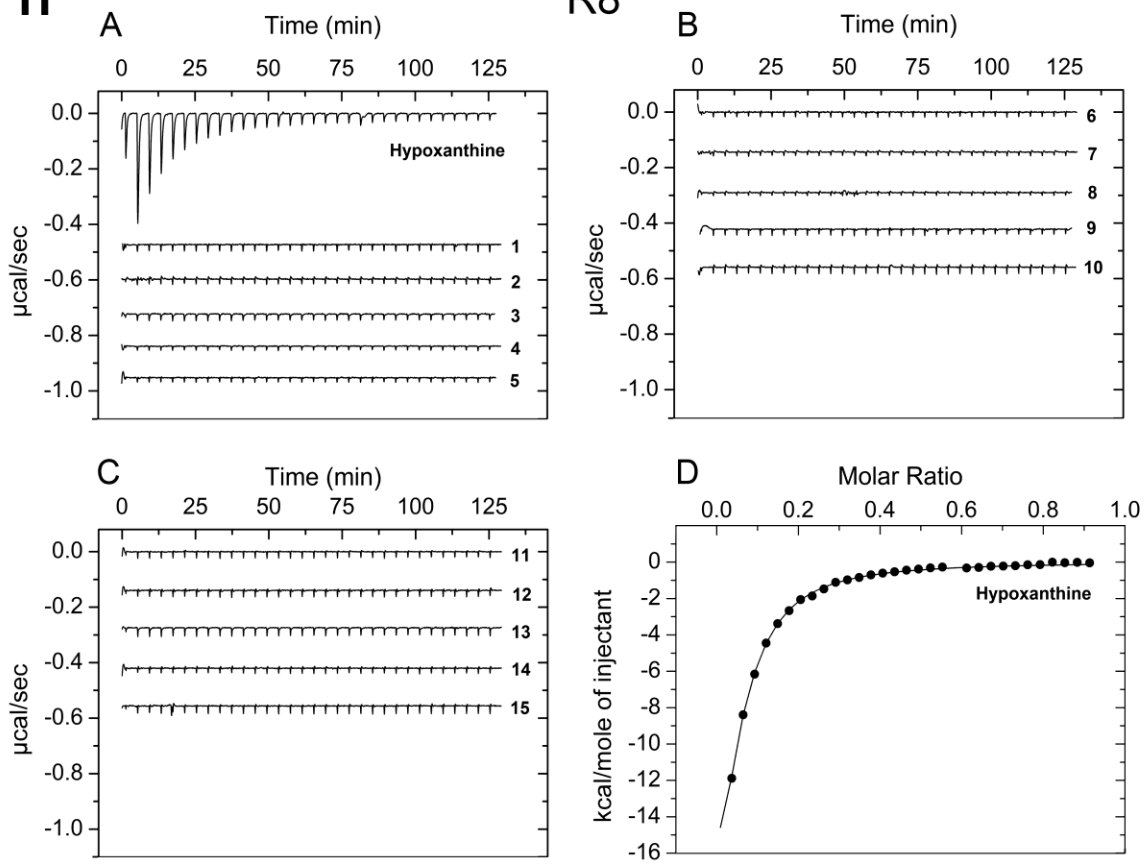
A
Time (min)
0 25 50 75 100 125
0.0 -0.2 -0.4 -0.6 -0.8 -1.0
µcal/sec
Hypoxanthine
1
2
3
4
5
B
Time (min)
0 25 50 75 100 125
0.0 -0.2 -0.4 -0.6 -0.8 -1.0
µcal/sec
6
7
8
9
10
C
Time (min)
0 25 50 75 100 125
0.0 -0.2 -0.4 -0.6 -0.8 -1.0
µcal/sec
11
12
13
14
15
D
Molar Ratio
0.0 0.2 0.4 0.6 0.8 1.0
0 -2 -4 -6 -8 -10 -12 -14 -16
kcal/mole of injectant
Hypoxanthine

III

R14

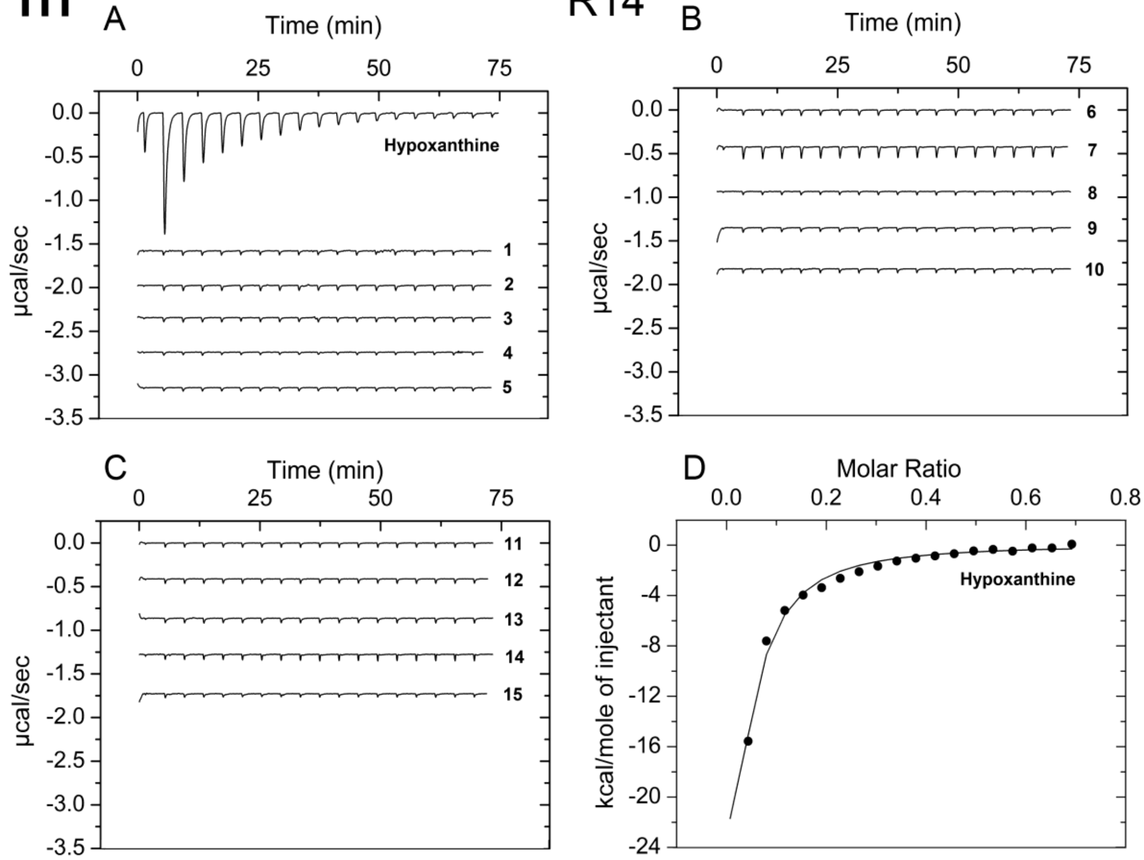
A
Time (min)
0 25 50 75
0.0 -0.5 -1.0 -1.5 -2.0 -2.5 -3.0 -3.5
µcal/sec
Hypoxanthine
1
2
3
4
5
B
Time (min)
0 25 50 75
0.0 -0.5 -1.0 -1.5 -2.0 -2.5 -3.0 -3.5
µcal/sec
6
7
8
9
10
C
Time (min)
0 25 50 75
0.0 -0.5 -1.0 -1.5 -2.0 -2.5 -3.0 -3.5
µcal/sec
11
12
13
14
15
D
Molar Ratio
0.0 0.2 0.4 0.6 0.8
0 -4 -8 -12 -16 -20 -24
kcal/mole of injectant
Hypoxanthine

Supplementary Fig. 12. Multiple sequence alignments and sequence logos of purine, amino acid and amine specific dCache_1 domains. The logos have been generated based on the protein sequences of amino acid and amine receptors reported previously [4,5] and the sequences of the purine responsive dCache_1 domains. Gproteobac: Gammaproteobacteria; Aproteobac: Alphaproteobacteria.

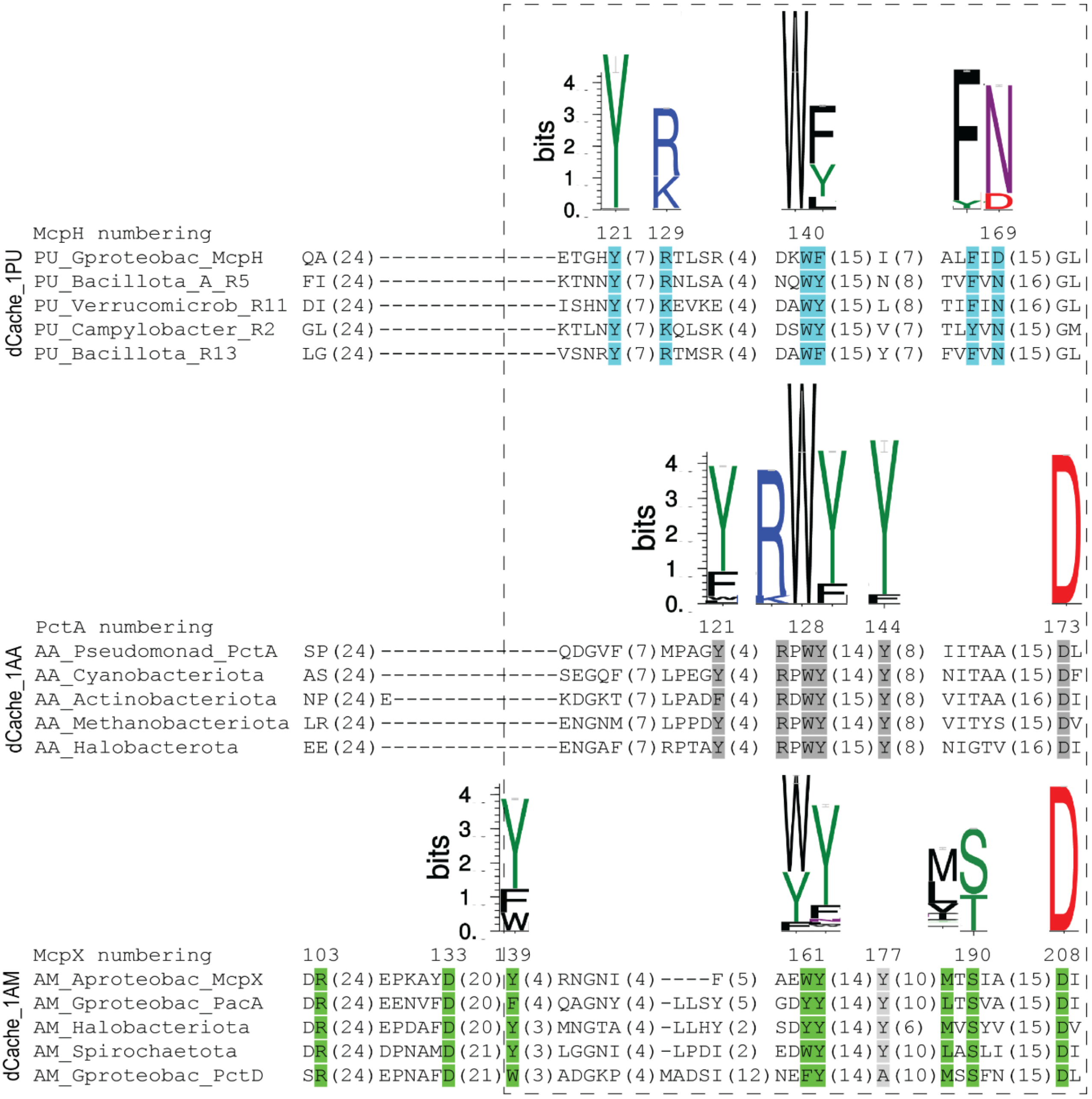

Supplementary Figure 13. Bayesian phylogenetic tree depicted in Figure 6 with proteins annotated.

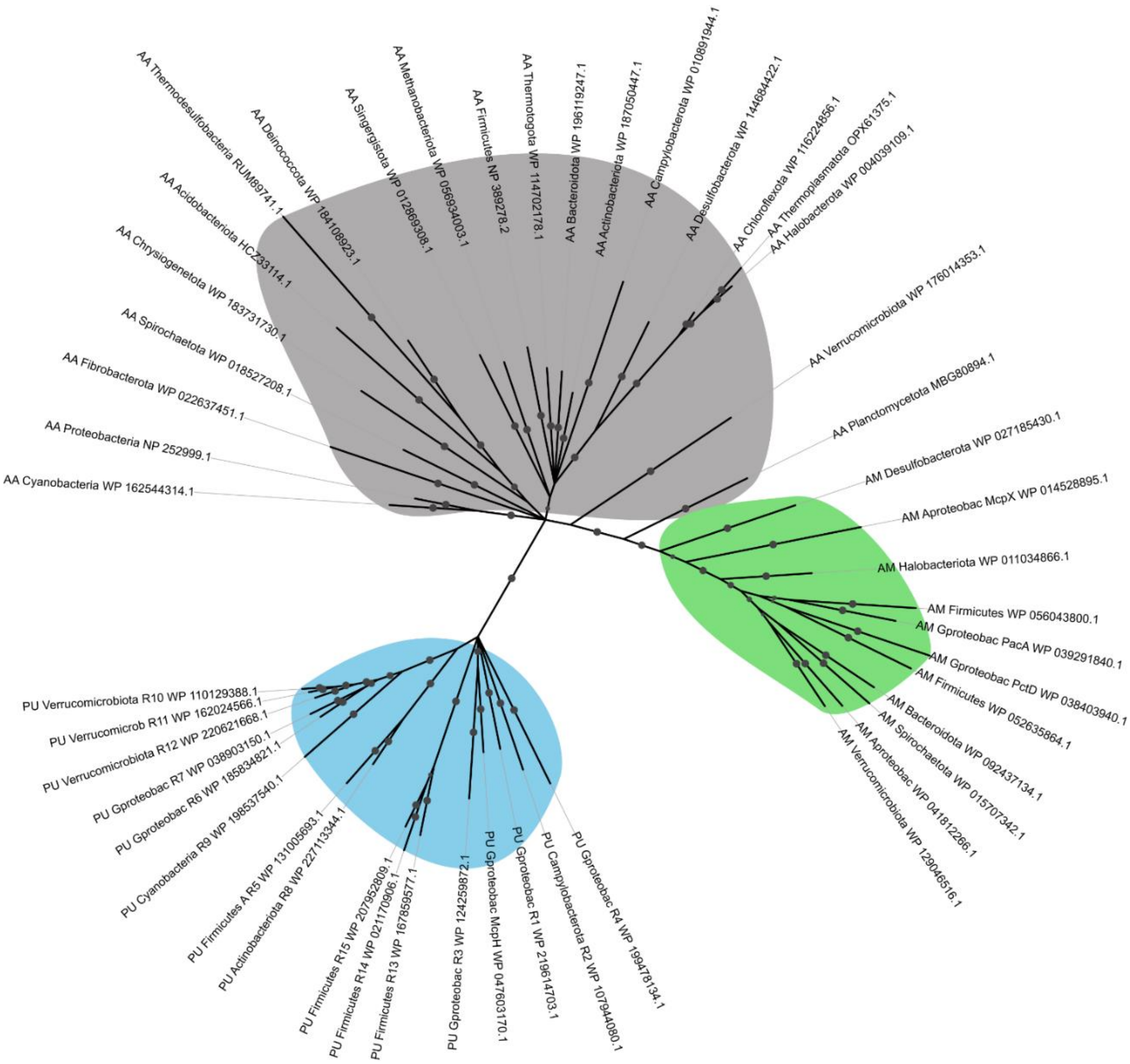

Supplementary Fig. 14. Guanosine modulates the activity of a diguanylate cyclase (R6) of *Vibrio cholerae*. Shown are colony morphology and c-di-GMP levels (green fluorescence) of *P. putida* harbouring plasmid pCdrA::*gfp*^{C} (c-di-GMP biosensor) and pBBR1MCS-2_START_R6 (R6 expression plasmid) in the absence and presence of increasing guanosine concentrations. The brightness of the colony morphology images has been adjusted to optimize visibility. Pictures were taken after 24 h of incubation at 30 ºC.

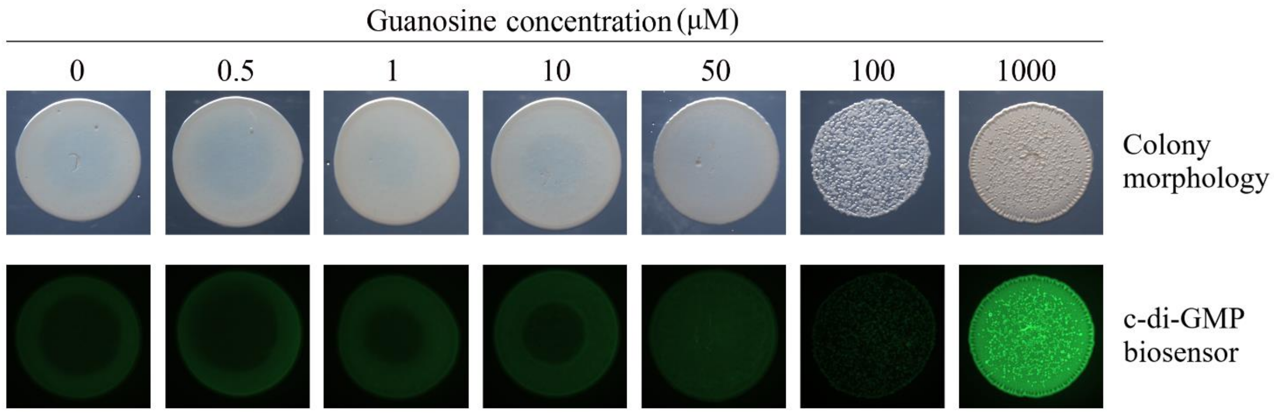

Supplementary Figure 15. Inosine, theophylline and guanosine do not alter colony morphology and c-di-GMP levels of *P. putida* containing the empty plasmid pBBR1MCS-2_START. A. Colony morphology and c-di-GMP levels (using c-di-GMP biosensor plasmid pCdrA::*gfp*^{C}) in the absence and presence of increasing inosine, theophylline and guanosine concentrations. The brightness of the colony morphology images has been adjusted to optimize visibility. **B.** c-di-GMP levels during growth in liquid cultures of *P. putida* in the absence and presence of different theophylline concentrations. Experiments were conducted under identical condition as those shown in Fig. 7 and Supplementary Fig. 13, except that the strain harbored the empty plasmid pBBR1MCS-2_START instead of the R6 expression plasmid pBBR1MCS-2_START_R6. RFU, relative fluorescence units; Abs, absorbance at 600 nm.

A

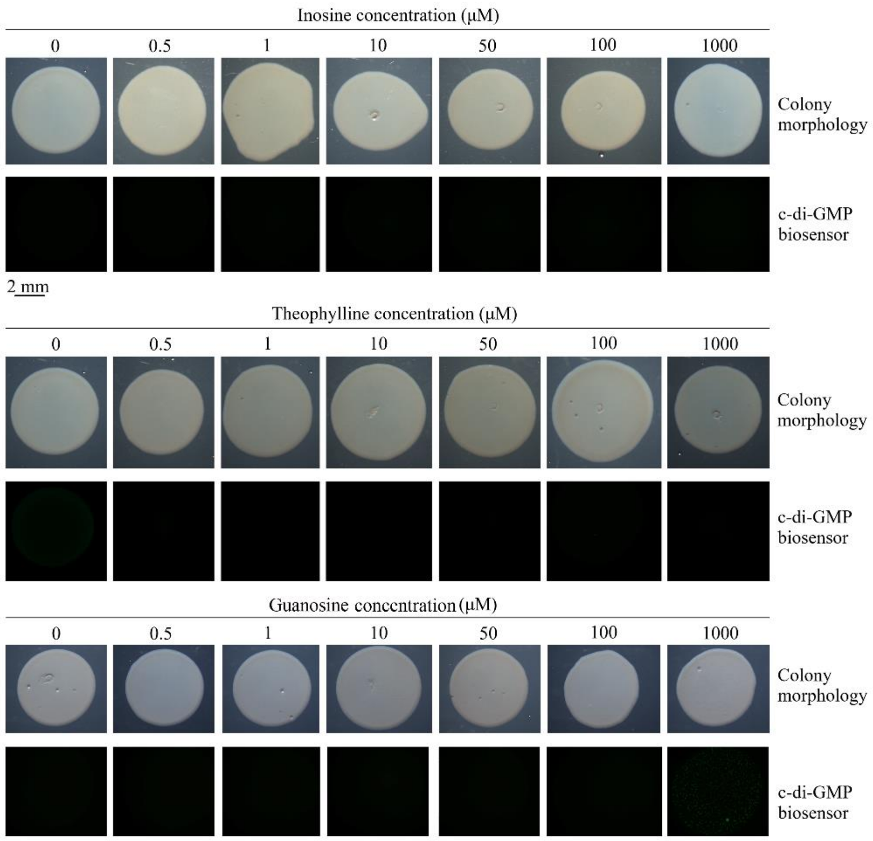

B

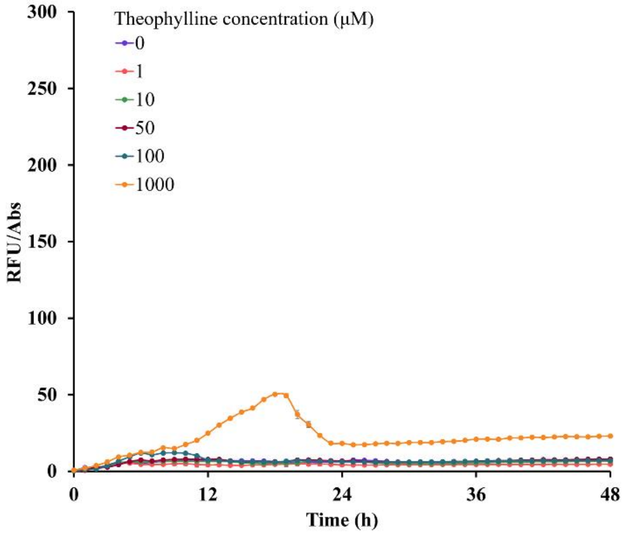

Supplementary Figure 16. Inosine and theophylline do not modulate the activity of the N164A mutant of the VC2224 diguanylate cyclase (R6) of *Vibrio cholerae*. N164 of VC2224 corresponds to D169 of McpH. Replacement of McpH D169 with alanine abolished the binding of adenine (Table 1, Supplementary Fig. 4). Colony morphology and c-di-GMP levels (green fluorescence) of *P. putida* harboring plasmid pCdrA::*gfp*^{C} (c-di-GMP biosensor) and pBBR1MCS-2_START_R6 N164A (expression plasmid for VC2224 N164A) in the absence and presence of increasing inosine and theophylline concentrations. The brightness of the colony morphology images has been adjusted to optimize visibility. Pictures were taken after 24 h of incubation at 30 ºC.

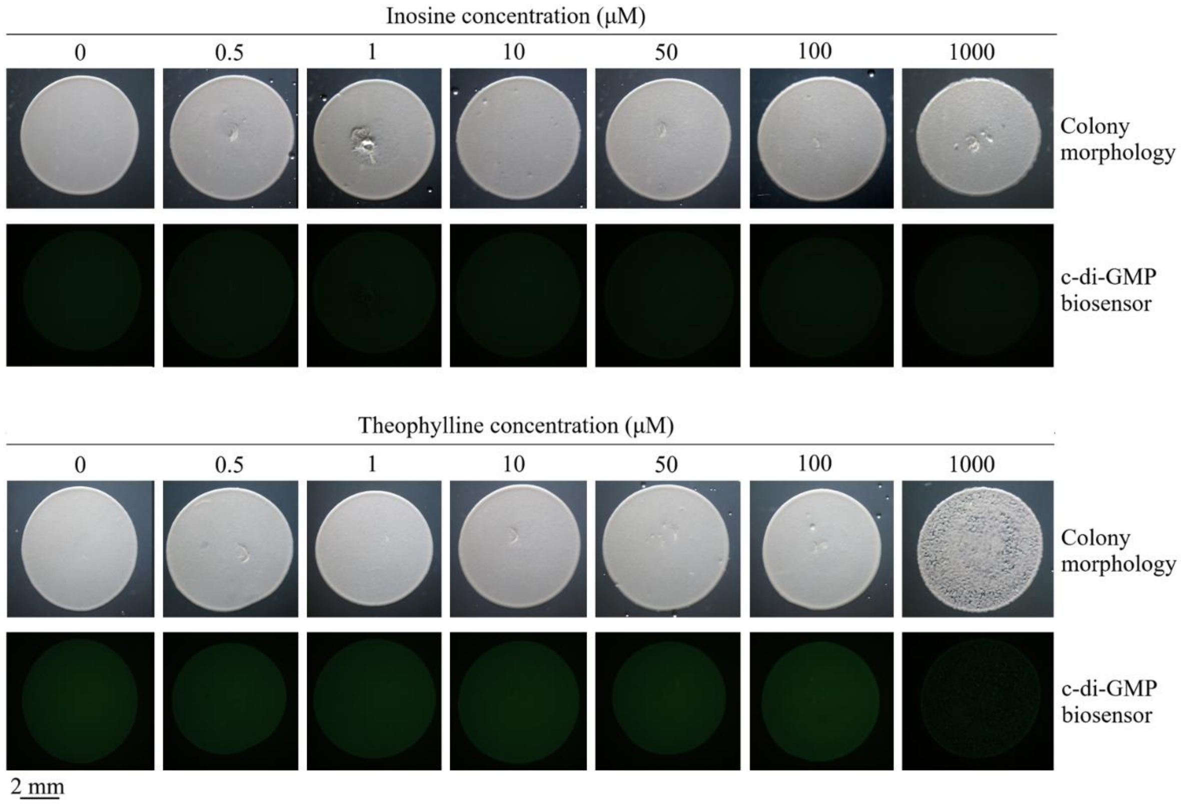

Supplementary Table 1. Structural alignment of McpH-LBD with all structures deposited in the protein data bank using the DALI [6] algorithm.

PDB ID	Acronym	Z-score	Rmsd[a] (Å)	Identity (%)	Species	Receptor family[b]	Bound ligand	Reference
3LIB	mmHK1S-Z3	19.1	2.9	15	*Methanosarcina mazei*	HK	-	[7]
7PRQ	PctD	18.8	3.0	16	*Pseudomonas aeruginosa*	CR	Choline	[8]
6MNI	PscC	18.6	3.1	16	*Pseudomonas syringae*	CR	Proline	[9]
5LT9	PctB	18.4	2.8	20	*P. aeruginosa*	CR	L-Arg	[10]
6FU4	TlpQ	18.3	3.2	17	*P. aeruginosa*	CR	Histamine	[11]
3LIF	rpHK1S-Z16	17.9	2.7	15	*Rhodopseudomonas palustris*	CR	-	[7]
6D8V	McpX	17.8	2.9	22	*Sinorhizobium meliloti*	CR	1,1-dimethyl-prolinium	[12]
6PZJ	-	17.7	3.0	14	*Leptospira interrogans*	CR	-	Unpublished
5ERE	-	17.2	2.9	11	*Desulfohalobium retbaense*	Stand-alone sensor domain	-	Unpublished
2ZBB	DctB	17.2	3.0	12	*Escherichia coli*	HK	Malonic acid	Unpublished
3BY9	DctB	17.2	3.3	13	*Vibrio cholerae*	HK	Succinic acid	[13]
3LIC	soHK1S-Z6	16.8	3.3	16	*Shewanella oneidensis*	HK	-	[7]
4WY9	Tlp1	16.1	3.1	12	*Campylobacter jejuni*	CR	-	[14]
6E0A	TlpA	16.0	3.1	12	*Helicobacter pylori*	CR	-	[15]
3LID	vpHK1S-Z8	15.7	3.8	12	*Vibrio parahaemolyticus*	HK	-	[7]
4XMQ	Tlp3 (CcmL)	15.0	3.2	13	*C. jejuni*	CR	-	[16]

[a]Rmsd, root mean square deviation.
[b]CR, chemoreceptor; HK, histidine kinase.

Supplementary Table 2. References for the lifestyle and pathogenicity of species harboring the 15 receptors studied (Table 2).

Name	Pathogenicity/characteristics	Refs.
R1	Fish pathogen, mainly salmon. Important economic losses	[17]
R2	Human pathogen, causes inflammatory bowel disease	[18]
R3	Human pathogen, causes lung infections. High mortality rate	[19]
R4	Halophyte plant isolate, can promote plant growth	[20]
R5	Human pathogen, at times fatal. Disrupts gut microbiota	[21]
R6	Human pathogen, causes life-threatening diarrheal disease	[22]
R7	Plant pathogen, in particular maize and rice	[23]
R8	Human pathogen, causes life-threatening bacteremia	[24]
R9	N_2-fixing bacterium, isolated from freshwater	[25]
R10	Isolated from a marine saltern	[26]
R11	Efficient degrader of fucoidan, a major algae product	[27]
R12	Isolated from deep sea cold seep	[28]
R13	N_2-fixing isolated from orchid roots	[29]
R14	Anaerobic, endospore forming bacterium, isolated from leafs	[30]
R15	Isolated from agricultural soil	[31]

Supplementary Table 3. N values derived from microcalorimetric titrations with different ligands. Dissociation constants of each protein and representative titration curves are shown in Table 3 and Fig. 4, respectively.

name	Adenine	Adenosine	Purine	Guanine	Guanosine	Inosine	Xanthine	Hypoxanthine	Uric acid	Theophylline	Caffeine	Cytosine	Thymine	Allantoin
	Purine compounds											Pyrimidine compounds		
R1	0.18*	0.13	Nts[b]	Nts[b]	0.21	0.12	Nb[a]	Nts[b]	Nts[b]	0.11*	Nts[b]	0.21*	0.20*	Nts[b]
R2	Nb[a]	Nb[a]	Nts[b]	Nts[b]	Nb[a]	Nb[a]	Nts[b]	0.21*	Nb[a]	0.33*	Nb[a]	0.32*	1.03*	Nts[b]
R3	Insoluble protein													
R4	0.32*	0.58*	0.18*	Nts[b]	Nts[b]	Nts[b]	Nts[b]	0.29*	Nts[b]	0.15*	Nb[a]	0.28*	0.14*	Nts[b]
R5	Nb[a]	Nts[b]	Nb[a]	Nts[b]	Nts[b]	Nts[b]	Nb[a]	0.02*	Nb[a]	0.10*	Nts[b]	Nts[b]	Nts[b]	Nts[b]
R6	Nts[b]	Nts[b]	Nts[b]	Nts[b]	0.35*	0.45*	Nts[b]	Nb[a]	Nts[b]	0.19*	Nts[b]	Nts[b]	Nts[b]	Nts[b]
R7	Insoluble protein													
R8	Nts[b]	Nts[b]	Nts[b]	Nts[b]	Nts[b]	0.12*	0.15*	0.05*	Nts[b]	0.14*	Nts[b]	Nts[b]	Nts[b]	Nb[a]
R9	Insufficient protein expression													
R10	Nts[b]	Nts[b]	Nts[b]	Nts[b]	TSA[c]	TSA[c]	TSA[c]	TSA[c]	Nts[b]	TSA[c]	Nts[b]	Nts[b]	Nts[b]	Nts[b]
R11	0.22*	Nb[a]	Nb[a]	Nts[b]	TSA[c]	0.16*	Nb[a]	Nb[a]	Nb[a]	0.15*	Nb[a]	Nb[a]	0.19*	Nb[a]
R12	Unfolded protein													
R13	Nts[b]	Nts[b]	Nts[b]	TSA[c]	Nts[b]	Nts[b]	Nts[b]	Nts[b]	Nts[b]	Nts[b]	Nts[b]	Nts[b]	Nts[b]	Nts[b]
R14	Nb[a]	0.41*	0.19*	Nts[b]	0.13*	0.09*	0.13*	0.02*	Nts[b]	0.04*	Nts[b]	0.46*	0.99*	Nb[a]
R15	Nts[b]	Nb[a]	Nb[a]	Nts[b]	0.70*	0.22*	0.05*	0.03*	Nb[a]	0.24*	Nb[a]	Nb[a]	Nb[a]	Nts[b]

[a] Nb: No binding observed in microcalorimetric titrations.

[b] Nts: No binding observed in thermal shift assays, i.e. compounds caused changes in the midpoint of protein unfolding (Tm) of less than 2 ºC.

[c] TSA: Binding observed in thermal shift assays, i.e. compounds caused changes in Tm by more than 2 ºC.

*Hyperbolic curve.

Supplementary Table 4. Presence of theophylline in different samples.

Sample type	Comment/reference
**Plants and plant extracts**	
Black tea	[32,33]
Coffee	[34,35]
Chocolate	[36]
Mate	[37]
Other plant products like lemon, pummelo or guaran	[38]
Bermuda grass	[39]
Citrus flowers (*Citrus limon*, *Citrus maxima, Citrus paradise*)	[40]
Different plants	[41]
Throughout the plant kingdom	[42]
**Human/animals**	
Haliotis discus hannai (mollusc)	[43]
Anopheles gambiae (insect)	[44]
Human	Many different tissues, cells and excretions [38]
Mouse	ST000696 of https://www.metabolomicsworkbench.org
Rat	ST000017 of https://www.metabolomicsworkbench.org
Baboon	ST002132 of https://www.metabolomicsworkbench.org
Goat	ST001968 of https://www.metabolomicsworkbench.org
Squirrel	ST000724 of https://www.metabolomicsworkbench.org
Pigs	[45]
**Microbiomes**	
Nasal microbiome	[46]
Cervical microbiota	[47]
Gut microbiome of insects	[48]
**Microorganisms**	
Escherichia coli	[49]
Staphylococcus aureus	[50]
Treponema pectinovorum	Project ST000692 of https://www.metabolomicsworkbench.org
Trypanosoma brucei	[51]

Supplementary Table 5. Strains, plasmids and oligonucleotides used.

Strains, plasmids and oligonucleotides	Genotype or relevant characteristics[a]	Ref.
**Strains**		
Escherichia coli BL21(DE3)	F^- *ompT gal dcm lon hsdS*$_B$($r_B^- m_B^-$) λ(DE3 [*lacI lacUV5-T7p07 ind1 sam7 nin5*]) [*malB*$^+$]$_{K-12}$(λ^S)	52
Pseudomonas putida KT2440	Wild type strain	53
**Plasmids**		
pET28_LBD_McpH	Km^R; pET28b(+) derivative containing the DNA fragment encoding McpH-LBD	3
pET28_LBD_McpH_Y121A	Km^R; pET28b(+) derivative containing the DNA fragment encoding McpH-LBD Y121A	This study[b]
pET28_LBD_McpH_R129A	Km^R; pET28b(+) derivative containing the DNA fragment encoding McpH-LBD R129A	This study[b]
pET28_LBD_McpH_W140A	Km^R; pET28b(+) derivative containing the DNA fragment encoding McpH-LBD W140A	This study[b]
pET28_LBD_McpH_F167A	Km^R; pET28b(+) derivative containing the DNA fragment encoding McpH-LBD F167A	This study[b]
pET28_LBD_McpH_D169A	Km^R; pET28b(+) derivative containing the DNA fragment encoding McpH-LBD D169A	This study[b]
pET28_LBD_McpH_D169N	Km^R; pET28b(+) derivative containing the DNA fragment encoding McpH-LBD D169N	This study[b]
pET28_WP_219614703-LBD	Km^R; pET28b(+) derivative containing a DNA fragment encoding WP_219614703-LBD (R1)	This study[b]
pET28_WP_107944080-LBD	Km^R; pET28b(+) derivative containing a DNA fragment encoding WP_107944080-LBD (R2)	This study[b]
pET28_WP_124259872-LBD	Km^R; pET28b(+) derivative containing a DNA fragment encoding WP_124259872-LBD (R3)	This study[b]
pET28_WP_199478134-LBD	Km^R; pET28b(+) derivative containing a DNA fragment encoding WP_199478134-LBD (R4)	This study[b]
pET28_WP_131005693-LBD	Km^R; pET28b(+) derivative containing a DNA fragment encoding WP_131005693-LBD (R5)	This study[b]
pET28_WP_185834821-LBD	Km^R; pET28b(+) derivative containing a DNA fragment encoding WP_185834821-LBD (R6)	This study[b]
pET28_WP_038903150-LBD	Km^R; pET28b(+) derivative containing a DNA fragment encoding WP_038903150-LBD (R7)	This study[b]
pET28_WP_227113344-LBD	Km^R; pET28b(+) derivative containing a DNA fragment encoding WP_227113344-LBD (R8)	This study[b]
pET28_WP_198537540-LBD	Km^R; pET28b(+) derivative containing a DNA fragment encoding WP_198537540-LBD (R9)	This study[b]
pET28_WP_110129388-LBD	Km^R; pET28b(+) derivative containing a DNA fragment encoding WP_110129388-LBD (R10)	This study[b]
pET28_WP_167859577-LBD	Km^R; pET28b(+) derivative containing a DNA fragment encoding WP_167859577-LBD (R13)	This study[b]
pET28_WP_021170906-LBD	Km^R; pET28b(+) derivative containing a DNA fragment encoding WP_021170906-LBD (R14)	This study[b]
pET28_WP_162024566-LBD	Km^R; pET28b(+) derivative containing a DNA fragment encoding WP_162024566-LBD (R11)	This study[b]
pET28_WP_207952809-LBD	Km^R; pET28b(+) derivative containing a DNA fragment encoding WP_207952809-LBD (R15)	This study[b]

pET28_WP_220621668-LBD	Km^R; pET28b(+) derivative containing a DNA fragment encoding WP_220621668-LBD (R12)	This study[b]
pBBR1MCS-2_START	Km^R; *oriRK2 mobRK2 lacZ*	[54]
pBBR1MCS-2_START_R6	Km^R; pBBR1MCS-2_START derivative containing full length coding sequence for WP_185834821 (R6) cloned into NdeI/BamHI sites	This study[b]
pBBR1MCS-2_START_R6 N164A[c]	Km^R; pBBR1MCS-2_START derivative containing the N164A mutant of the full length coding sequence for WP_185834821 (R6) cloned into NdeI/BamHI sites	This study
pCdrA::*gfp*[c]	Ap^R, Gm^R; FleQ-dependent c-di-GMP biosensor	[55]
**Oligonucleotides (5'-3')**		
WP_185834821-NdeI-F	GGAAACACATATGACCCGTAAGAAATTC	This study
N164A_R	CGAAAACTTTGTAGGCCACGAAAACCACGG	This study
N164A_F	CCGTGGTTTTCGTGGCCTACAAAGTTTTCG	This study
WP_185834821-BamHI-R	CTAGTGGATCCTTACAGGCTAACC	This study

[a]Ap, ampicillin; Km, kanamycin; Gm, gentamicin.
[b]Gene synthesis and plasmid construction were done by GenScript Inc.
[c]N164 corresponds to McpH N169.

Supplementary Table 6. Buffers used for the analysis of the recombinant proteins used in this study. Buffers were chosen to optimize protein solubility.

Protein name	Analysis buffer
McpH-LBD	3 mM Tris, 3 mM PIPES, 3 mM MES, pH 6.0
McpH-LBD Y121A	3 mM Tris, 3 mM PIPES, 3 mM MES, pH 6.0
McpH-LBD R129A	3 mM Tris, 3 mM PIPES, 3 mM MES, pH 6.0
McpH-LBD W140A	3 mM Tris, 3 mM PIPES, 3 mM MES, pH 6.0
McpH-LBD F167A	3 mM Tris, 3 mM PIPES, 3 mM MES, pH 6.0
McpH-LBD D169A	3 mM Tris, 3 mM PIPES, 3 mM MES, pH 6.0
McpH-LBD D169N	3 mM Tris, 3 mM PIPES, 3 mM MES, pH 6.0
WP_219614703-LBD (R1)	3 mM Tris, 3 mM PIPES, 3 mM MES, 150 mM NaCl, 10 % (v/v) glycerol, pH 7.0
WP_107944080-LBD (R2)	3 mM Tris, 3 mM PIPES, 3 mM MES, 150 mM NaCl, 10 % (v/v) glycerol, pH 8.0
WP_124259872-LBD (R3)	Insoluble
WP_199478134-LBD (R4)	3 mM Tris, 3 mM PIPES, 3 mM MES, 150 mM NaCl, 10 % (v/v) glycerol, pH 8.0
WP_131005693-LBD (R5)	3 mM Tris, 3 mM PIPES, 3 mM MES, 150 mM NaCl, 10 % (v/v) glycerol, pH 7.0
WP_185834821-LBD (R6)	3 mM Tris, 3 mM PIPES, 3 mM MES, 150 mM NaCl, 10 % (v/v) glycerol, pH 8.0
WP_038903150-LBD (R7)	Insoluble
WP_227113344-LBD (R8)	3 mM Tris, 3 mM PIPES, 3 mM MES, 150 mM NaCl, 10 % (v/v) glycerol, 1 mM β-mercaptoethanol, pH 6.0
WP_198537540-LBD (R9)	Insufficient protein expression
WP_110129388-LBD (R10)	3 mM Tris, 3 mM PIPES, 3 mM MES, 150 mM NaCl, 10 % (v/v) glycerol, 1 mM β-mercaptoethanol, pH 7.0
WP_162024566-LBD (R11)	3 mM Tris, 3 mM PIPES, 3 mM MES, 150 mM NaCl, 10 % (v/v) glycerol, 1 mM β-mercaptoethanol, pH 7.0
WP_220621668-LBD (R12)	3 mM Tris, 3 mM PIPES, 3 mM MES, 150 mM NaCl, 10 % (v/v) glycerol, pH 7.5
WP_167859577-LBD (R13)	3 mM Tris, 3 mM PIPES, 3 mM MES, 150 mM NaCl, 10 % (v/v) glycerol, pH 7.0
WP_021170906-LBD (R14)	Unfolded protein
WP_207952809-LBD (R15)	3 mM Tris, 3 mM PIPES, 3 mM MES, 150 mM NaCl, 10 % (v/v) glycerol, pH 7.0

Supplementary Table 7. Sequences of recombinant proteins used in this study. The hexa-histidine containing extension (underlined) and mutations to alanine or asparagine are shown in bold face.

Protein name	Protein sequence (including his-tag containing extension)
McpH-LBD	**MGSSHHHHHHSSGLVPRGSHM**NRLTDRYLVDTALPASIEAIRNDIERMLG<br>QPLVAAADIAGNTLLRDWLAAGEDPAQAPQFIEYLTAAKQRNHAFTTLFA<br>STETGHYYNENGLDRTLSRSNPKDKWFYGYIDSGAERFINIDIDGATGEL<br>ALFIDYRVEKEGKLVGVAGMGLRMTELSKLIHDFSFGEHGKVFLVRNDGL<br>IQVHPDAAFSGKRQLAEQLGADAAKGVMTGGESLRSSRFSRDGERYLALG<br>LPLRDLNWTLVAEVPESEIYAQMHQ
McpH-LBD Y121A	**MGSSHHHHHHSSGLVPRGSHM**NRLTDRYLVDTALPASIEAIRNDIERMLG<br>QPLVAAADIAGNTLLRDWLAAGEDPAQAPQFIEYLTAAKQRNHAFTTLFA<br>STETGH**A**YNENGLDRTLSRSNPKDKWFYGYIDSGAERFINIDIDGATGEL<br>ALFIDYRVEKEGKLVGVAGMGLRMTELSKLIHDFSFGEHGKVFLVRNDGL<br>IQVHPDAAFSGKRQLAEQLGADAAKGVMTGGESLRSSRFSRDGERYLALG<br>LPLRDLNWTLVAEVPESEIYAQMHQ
McpH-LBD R129A	**MGSSHHHHHHSSGLVPRGSHM**NRLTDRYLVDTALPASIEAIRNDIERMLG<br>QPLVAAADIAGNTLLRDWLAAGEDPAQAPQFIEYLTAAKQRNHAFTTLFA<br>STETGHYYNENGLD**A**TLSRSNPKDKWFYGYIDSGAERFINIDIDGATGEL<br>ALFIDYRVEKEGKLVGVAGMGLRMTELSKLIHDFSFGEHGKVFLVRNDGL<br>IQVHPDAAFSGKRQLAEQLGADAAKGVMTGGESLRSSRFSRDGERYLALG<br>LPLRDLNWTLVAEVPESEIYAQMHQ
McpH-LBD W140A	**MGSSHHHHHHSSGLVPRGSHM**NRLTDRYLVDTALPASIEAIRNDIERMLG<br>QPLVAAADIAGNTLLRDWLAAGEDPAQAPQFIEYLTAAKQRNHAFTTLFA<br>STETGHYYNENGLDRTLSRSNPKDK**A**FYGYIDSGAERFINIDIDGATGEL<br>ALFIDYRVEKEGKLVGVAGMGLRMTELSKLIHDFSFGEHGKVFLVRNDGL<br>IQVHPDAAFSGKRQLAEQLGADAAKGVMTGGESLRSSRFSRDGERYLALG<br>LPLRDLNWTLVAEVPESEIYAQMHQ
McpH-LBD F167A	**MGSSHHHHHHSSGLVPRGSHM**NRLTDRYLVDTALPASIEAIRNDIERMLG<br>QPLVAAADIAGNTLLRDWLAAGEDPAQAPQFIEYLTAAKQRNHAFTTLFA<br>STETGHYYNENGLDRTLSRSNPKDKWFYGYIDSGAERFINIDIDGATGEL<br>AL**A**IDYRVEKEGKLVGVAGMGLRMTELSKLIHDFSFGEHGKVFLVRNDGL<br>IQVHPDAAFSGKRQLAEQLGADAAKGVMTGGESLRSSRFSRDGERYLALG<br>LPLRDLNWTLVAEVPESEIYAQMHQ
McpH-LBD D169A	**MGSSHHHHHHSSGLVPRGSHM**NRLTDRYLVDTALPASIEAIRNDIERMLG<br>QPLVAAADIAGNTLLRDWLAAGEDPAQAPQFIEYLTAAKQRNHAFTTLFA<br>STETGHYYNENGLDRTLSRSNPKDKWFYGYIDSGAERFINIDIDGATGEL<br>ALFI**A**YRVEKEGKLVGVAGMGLRMTELSKLIHDFSFGEHGKVFLVRNDGL<br>IQVHPDAAFSGKRQLAEQLGADAAKGVMTGGESLRSSRFSRDGERYLALG<br>LPLRDLNWTLVAEVPESEIYAQMHQ
McpH-LBD D169N	**MGSSHHHHHHSSGLVPRGSHM**NRLTDRYLVDTALPASIEAIRNDIERMLG<br>QPLVAAADIAGNTLLRDWLAAGEDPAQAPQFIEYLTAAKQRNHAFTTLFA<br>STETGHYYNENGLDRTLSRSNPKDKWFYGYIDSGAERFINIDIDGATGEL<br>ALFI**N**YRVEKEGKLVGVAGMGLRMTELSKLIHDFSFGEHGKVFLVRNDGL<br>IQVHPDAAFSGKRQLAEQLGADAAKGVMTGGESLRSSRFSRDGERYLALG<br>LPLRDLNWTLVAEVPESEIYAQMHQ
WP_219614703-LBD (R1)	**MGSSHHHHHHSSGLVPRGSHM**QRSAQQLIETRMFEQELPNLTQRIGKEIE<br>KDLTSVANAARQLANDRFVLDWVARGMPKEQESILIDQLKDMTAQYGLVT<br>ASFADRQSAAYYNQDGFLRNLTPEQDAWFYGYTKSPQDLMLSIFRETNGE<br>VKLFVNFQQLNGRGLAGLAKSLDSMVSMLANFRIGDSGFVFMTDGSGKVK<br>LHPDAARIDRDNLTQLASGTTANLLTKQAFAATQAEVDGQAVILATSYIP<br>MLDWYLVAQVPEAEIYAELDKARLH
WP_107944080-LBD (R2)	**MGSSHHHHHHSSGLVPRGSHM**NLYTEKVVKDELPLAVSNVAGEIGYAIDK<br>IINTSYQMTKNDYLLKWIDEGEPKDGLATLFNYNTDLMKAFNLSTAMFVS<br>DKTLNYYTNDKILKQLSKDNPRDSWYFDVKNGKEVNSLNIQVSEATGSLT

	LYVNSKVEKDGKFYGVSAIGMNLDDIVNLVTSKTMGEGSKFLMVDSSGIV<br>KIEKSDRVGKVNVKDVLGKEKFDVLMNKNGGVIRHFNGTRNLIIGSKYIP<br>SLDWYLFGEMDEDVLLKDLHT
WP_124259872-LBD (R3)	**MGSSHHHHHHSSGLVPRGSHM**KERAREQDLPTALGEIRSEVLRQIAAPVA<br>LTRSLATNEYILNWEEQGLPEQGAAAWKTYAQTLKNESHAATIAWVSEKT<br>GKYLDENGFSRTVQRSDATDNWFYDFLSKGKQLEIKLGKDKASSVYNLFI<br>DARFDVNGKIGVAALGLSVNELADFIRKQKIGNSGFVYLVSPDGAFVIHR<br>DAALADGQHFLRDTSGFNQEMVTKLLMGQRFSSVSYSATDGERIAAASYV<br>PELNLYIVAEVPQAEILGKITQT
WP_199478134-LBD (R4)	**MGSSHHHHHHSSGLVPRGSHM**MESDLKQLKEELLPNRLHSLSSRISEQIS<br>PLINASKLMTNDRFIADWVKKGADESRLPLVAEELNSIKQLSGSDSTFYV<br>VNMKSGLEFLGYDQKFFRTPLADYPYKEFYPNFLAKNKDYELNLQYADQK<br>LYINYRSREMDPTTGKPLVVAGLAIKVDKLIDMVKQLTIGKSGRAMLVTD<br>QGVIQAKGESPAIDLIKQTDIASLLQDKNQVQIVEKSISGKDYYLGALWV<br>PMLDRFIVIEVPSEQILSPIYQQ
WP_131005693-LBD (R5)	**MGSSHHHHHHSSGLVPRGSHM**GYQSNTNIFKNDIEHVSTLAAEGIYYQID<br>KLLSEPINVSLTMANDSLLKNFLDGEKEHLNDEEFIYKLQDYLNVYRNKY<br>SYDSVFLVSTKTNNYYHFNGLDRNLSANNSENQWYYTFLKNDDEYSLNVD<br>NDEASNNSITVFVNCKIKDDNGATMGIIGVGLKVNSLQMLLKGYNDKFDV<br>VARLIDDKGFVQLAVDKTGHENINFFENSSSDLSNSKQLILNNKKEQKSF<br>WYSSEKSKSYIVCQYIPSLKWHLVLENDFTLMIKQLHLQ
WP_185834821-LBD (R6)	**MGSSHHHHHHSSGLVPRGSHM**HDTLEEQINKDSLPLTSDNIYSEIQQDLI<br>RPIFISSLMAQDTFVREWTLAGEQDPERIIRYLREIQRQYQTISSFYISD<br>RTGHYYHYTGILKQVSESNPNDAWFYRVKNSAPDKNFEVNIDIDTANSQQ<br>TVVFVNYKVFDFENRFLGVIGVGLSSDAVSALVEKYQKRYNRHIYFINEL<br>GEVTLHGSHHPGFDQIQQREGLKTLATQILTSPSVGASYYADGQKVYLNS<br>RWVDEFQWYIIVEQKDEFNHD
WP_038903150-LBD (R7)	**MGSSHHHHHHSSGLVPRGSHM**ARHSLFDEISESSLPLTSDNVYSEIQRDL<br>LNPIFISSLMAHDTFVKDWVLSNETDPQAMTRYLREIDRRFNTVVSFFVS<br>NNTHRYYDPEKISHTLLETSPEDKWFFDIRDEKDGDPYDIEIGVDPENRT<br>RMDIFINYKVFDYSGNFIGVTGVGLPVQRVTQLIETYEQRYNRTIYLIDE<br>DGDVMLHSKAFHRASNIHQQPGLQSLATQVLTSPGGSYRYSLNGENIFLN<br>TRVIPEFGWKLMVEQNSGPHDRQLWLTLLKN
WP_227113344-LBD (R8)	**MGSSHHHHHHSSGLVPRGSHM**GYQSSRAAFEKDAERTSLLAAEGAAREID<br>NRFAEPIDVSLAMAHDTLLADLLSAEPTRGDDEAYADAVCAYLESYREAF<br>GFDSVFLVSTESNRYYHFNGVDRTLERDNPENTWYFDFLDRDEAYSLNVD<br>NDEATADEITMFVNARILDEQGSTLAIVGVGFRMDDLKELLAGFEARTDT<br>RVRLLADDGTIRASTDPNENGHALFDTEEAAVLSEQTRSDRTDVQDFWYR<br>ANGENGFLVSRYLPNLDWFLVVDHDTSQLDAQMARQF
WP_198537540-LBD (R9)	**MGSSHHHHHHSSGLVPRGSHM**ANALAAARRQVVESTLPLTLDALSADLQQ<br>DFVQPILFASAMAANTLLIDWVEQGEQPEAAVQRYLSRVQAQHGATTVFF<br>VSEASRRYYHPTGILKTVSPGSAQDAWFFRLRASASSYEVNLDRDTADPS<br>RTTVFVNYKLLGDGGRFLGAVGLGRSTSQLTRRIQQAERTNGIQVMFLDG<br>RGRILFSPRRGQAPPQLQRAIAQQRLRDSLREQPRGAFQFRQGGELIYVR<br>TKRIPELNWTLVVSQPLRVPSGPLWS
WP_110129388-LBD (R10)	**MGSSHHHHHHSSGLVPRGSHM**VSRNNVRKTLAESTLPLTSDNVYSEIQRD<br>LLRPVFIASLMANDTFLRDWAIAGEKDRDAIVRYLHEIKIKYGTVSSFFV<br>SDKTLKYYYAHGLLKTVSEDEPRDEWYFRVREMDEPYEINVDPDMANQDA<br>LTIFINYRVRDYAGNFIGATGVGLTVTKVNRLISRYEAKYDRQIYFVDAS<br>GNVVLRPSNSTMRGYDSLQEIEGLGERVADLLAGKTDSLTYKRLGDRRIM<br>NCRYVPELDWYLIVEQSEATMMAPLRQE
WP_162024566-LBD (R11)	**MGSSHHHHHHSSGLVPRGSHM**RSNMRLSITESTLPLTSDNVYSEIQRDLL<br>RPIFISSLMANDTFLRDWALNGEVDINQITKFLHEIKVEYSTISSFFVSD<br>ISHNYYHAQGLLKEVKENEPRDAWYFKARDMDAPYEINVDLDMANQDQMT<br>IFINYRVFDYNDNFIGMTGVGLTVKNVNNLISHYEAKYQRQIYFLNKDGE

	IVLRPSNSPLMNYQSLQNIPGLNDVSTDLLAQERTTLTYEREGTTYMLNC<br>RFIPELNWFLIIEQSEQELLAPIKEQ
WP_220621668-LBD (R12)	**MGSSHHHHHHSSGLVPRGSHM**SLPLTADNIYSVIQRDLLRPIFISSMMAN<br>DAFLRDWTINGEVDVKQMQRYLEEIRREYGTVTSFFISEKSRNYYYWGGV<br>LKQVDENEPRDVWYFRVREMEKPFEINVDIDMANNDALTVFVNYRVYDYE<br>GNYIGAAGTGLTVNRVNALIEEYEGRFNREIFFVDREGNIILGPSKGRLT<br>TYGNLDAVPGLKKDAQSLTSGTEEQKIRYERDGKTYFLNSRWIPELNWYL<br>MV
WP_167859577-LBD (R13)	**MGSSHHHHHHSSGLVPRGSHM**TQREAVDKLKTKDLLHLADSIAAKVDGQI<br>RKAKETSLLMAEDPNVINWVAGGERDEALGAVVGRKLTLLTSEHGYDNSF<br>LVSAVSNRYWGEGGTVLRTMSRSDPEDAWFYANVDNRKPIEIVLDYAIAR<br>GDTFVFVNALIGGAERALGETGVGLSLKQSAEQFQQFKYGDKSHLWLVDR<br>EGTIYLSDRYEQAGTKLNTILPGAVSDELFAGFANTQVVTYRTAQTGTID<br>LISRPIESADLDIVFAIPRSESVPFLHKIRTNT
WP_021170906-LBD (R14)	**MGSSHHHHHHSSGLVPRGSHM**THNAMVDKLKNRDMLYIVQSMSEKIDGRI<br>ERAQETSLLLADDPTVLAWVESGGRDEAAGEIVKTKITDIGKNYDYVKAF<br>VASTVTNQYWEDNKVVKPLSKTSYTDKWFYKALKSGKKIELNIDYDSIHN<br>ETFIFFNTLVGDVRQPVAVAGVALSLGDIAKEFGSYKFGEHSNLWLVDKQ<br>GKIHLADDLEYNGRLAGDFLPAEVMQQILGDMDNATARPKVLEYQDSQGR<br>IMDLAYQSTATTDWKLVFQIPRSESIAILSSVK
WP_207952809-LBD (R15)	**MGSSHHHHHHSSGLVPRGSHM**EKEVVNKLKSKDLVRIAESIASKIDGRLQ<br>RAQESSLTMAMDPELIEWLASGEKDQIAGAHVLQKLDNLAKGFDYSNGFI<br>ASRMTGSYWIEGKQMSKILSQTNPADKWFYDSFASQKRMALNIDYNDSRR<br>DTFVFVNVLVGDVESPVGVAGIGLSLKELANNFTNYKYGANSNVWLVDKS<br>GKIYLSDRVEDIGAMISSFVPEEAFEQMLGQLNETDSGLAKPVVLDYEDQ<br>EGKQMDLISYPLKSTDWKLLFQMRRSESVAFLDTIKLN

Supplementary Table 8. Data collection and refinement statistics. Statistics for the highest-resolution shell are shown in parentheses.

**Ligand**	**Uric Acid**
PDB ID	8BMV
Beam Line	XALOC (ALBA)
Space group	P 1 21 1
Unit cell a, b, c (Å)	32.581 124.322 57.201
Resolution (Å)	32.15 - 1.95 (2.02 - 1.95)
Unique reflections	31376 (3258)
Multiplicity	2.9 (3.1)
Completeness (%)	96.01 (99.48)
I/σ_I	13.07 (1.73)
Wilson B-factor	36.6
R_{merge} (%)	5.17 (70.7)
CC(1/2)	0.998 (0.678)
CC	1 (0.899)
**Refinement**	
R_{work}/R_{free} (%)	18.27 / 23.12
CC_{work}/CC_{free}	0.97 / 0.91
No. atoms	4177
Protein	4037
Ligands	24
Solvent	116
B-factor (Å^2)	47.50
R.m.s deviations	
Bond lengths (Å)	0.009
Bond angles (°)	1.01
Ramachandran (%)	
Favoured (%)	97.55
Outliers (%)	0.00

Supplementary References

1. Wallace, A. C., Laskowski, R. A. & Thornton, J. M. LIGPLOT: a program to generate schematic diagrams of protein-ligand interactions. *Prot. Engineer.* **8**, 127–34 (1995).
2. Oren, A. & Garrity, G. M. Valid publication of the names of forty-two phyla of prokaryotes. *Int. J. Syst. Evol. Microbiol.* **71**, (2021).
3. Fernández, M., Morel, B., Corral-Lugo, A. & Krell, T. Identification of a chemoreceptor that specifically mediates chemotaxis toward metabolizable purine derivatives. *Mol. Microbiol.* **99**, 34–42 (2016).
4. Gumerov, V. M. *et al.* Amino acid sensor conserved from bacteria to humans. *Proc. Natl. Acad. Sci. USA* **119**, e2110415119 (2022).
5. Cerna-Vargas, J. P., Gumerov, V. M., Krell, T. & Zhulin, I. B. Amine-recognizing domain in diverse receptors from bacteria and archaea evolved from the universal amino acid sensor. *Proc. Natl. Acad. Sci. USA* **120**, e2305837120 (2023).
6. Holm, L. & Rosenstrom, P. Dali server: conservation mapping in 3D. *Nucleic Acids Res.* **38**, W545-9 (2010).
7. Zhang, Z. & Hendrickson, W. A. Structural characterization of the predominant family of histidine kinase sensor domains. *J. Mol. Biol.* **400**, 335–53 (2010).
8. Matilla, M. A. *et al.* Chemotaxis of the Human Pathogen *Pseudomonas aeruginosa* to the Neurotransmitter Acetylcholine. *mBio* **13**, e0345821 (2022).
9. Ehrhardt, M. K. G., Gerth, M. L. & Johnston, J. M. Structure of a double CACHE chemoreceptor ligand-binding domain from *Pseudomonas syringae* provides insights into the basis of proline recognition. *Biochem. Biophys. Res. Commun.* **549**, 194–199 (2021).
10. Gavira, J. A. *et al.* How Bacterial Chemoreceptors Evolve Novel Ligand Specificities. *mBio* **11**, e03066-19 (2020).
11. Corral-Lugo, A. *et al.* High-Affinity Chemotaxis to Histamine Mediated by the TlpQ Chemoreceptor of the Human Pathogen *Pseudomonas aeruginosa*. *mBio* **9**, e01894-18 (2018).
12. Shrestha, M. *et al.* Structure of the sensory domain of McpX from *Sinorhizobium meliloti*, the first known bacterial chemotactic sensor for quaternary ammonium compounds. *Biochem. J.* **475**, 3949–3962 (2018).
13. Cheung, J. & Hendrickson, W. A. Crystal structures of C4-dicarboxylate ligand complexes with sensor domains of histidine kinases DcuS and DctB. *J. Biol. Chem.* **283**, 30256–65 (2008).
14. Machuca, M. A., Liu, Y. C., Beckham, S. A., Gunzburg, M. J. & Roujeinikova, A. The crystal structure of the tandem-PAS sensing domain of *Campylobacter jejuni* chemoreceptor Tlp1 suggests indirect mechanism of ligand recognition. *J. Struct. Biol.* **194**, 205–13 (2016).

15. Sweeney, E. G., Perkins, A., Kallio, K., James Remington, S. & Guillemin, K. Structures of the ligand-binding domain of *Helicobacter pylori* chemoreceptor TlpA. *Protein Sci.* **27**, 1961–1968 (2018).
16. Liu, Y. C., Machuca, M. A., Beckham, S. A., Gunzburg, M. J. & Roujeinikova, A. Structural basis for amino-acid recognition and transmembrane signalling by tandem Per-Arnt-Sim (tandem PAS) chemoreceptor sensory domains. *Acta crystallogr. D Biol. Crystallogr.* **71**, 2127–36 (2015).
17. Pradhan, S. K. *et al.* Isolation of Aeromonas salmonicida subspecies salmonicida from aquaculture environment in India: Polyphasic identification, virulence characterization, and antibiotic susceptibility. *Microb. Pathog.* **179**, 106100 (2023).
18. Zhang, L. *et al.* Bacterial Species Associated With Human Inflammatory Bowel Disease and Their Pathogenic Mechanisms. *Front. Microbiol.* **13**, 801892 (2022).
19. Fu, H. *et al.* Rapid detection of *Burkholderia cepacia* complex carrying the 16S rRNA gene in clinical specimens by recombinase-aided amplification. *Front. Cell. Infect. Microbiol.* **12**, 984140 (2022).
20. Lucena, T. *et al. Marinomonas spartinae* sp. nov., a novel species with plant-beneficial properties. *Int. J. Syst. Evol. Microbiol.* **66**, 1686–1691 (2016).
21. Markovska, R., Dimitrov, G., Gergova, R. & Boyanova, L. *Clostridioides difficile*, a New ‘Superbug’. *Microorganisms* **11**, 845 (2023).
22. Montero, D. A. *et al. Vibrio cholerae*, classification, pathogenesis, immune response, and trends in vaccine development. *Front. Med.* **10**, 1155751 (2023).
23. Hugouvieux-Cotte-Pattat, N. & Van Gijsegem, F. Diversity within the *Dickeya zeae* complex, identification of *Dickeya zeae* and *Dickeya oryzae* members, proposal of the novel species *Dickeya parazeae* sp. nov. *Int. J. Syst. Evol. Microbiol.* **71**, (2021).
24. Lau, S. K. P. *et al. Eggerthella hongkongensis* sp. nov. and *eggerthella sinensis* sp. nov., two novel Eggerthella species, account for half of the cases of *Eggerthella* bacteremia. *Diagn. Microbiol. Infect. Dis.* **49**, 255–263 (2004).
25. Di Cesare, A. *et al.* Genome analysis of the freshwater planktonic *Vulcanococcus limneticus* sp. nov. reveals horizontal transfer of nitrogenase operon and alternative pathways of nitrogen utilization. *BMC Genomics* **19**, 259 (2018).
26. Zhou, L.-Y., Wang, N.-N., Mu, D.-S., Liu, Y. & Du, Z.-J. *Coraliomargarita sinensis* sp. nov., isolated from a marine solar saltern. *Int. J. Syst. Evol. Microbiol.* **69**, 701–707 (2019).
27. Sichert, A. *et al. Verrucomicrobia* use hundreds of enzymes to digest the algal polysaccharide fucoidan. *Nat. Microbiol.* **5**, 1026–1039 (2020).

28. BioSample Ruficoccus sp. ZRK36. *https://www.ncbi.nlm.nih.gov/biosample/?term=Ruficoccus+sp.+ZRK36*.
29. Sun, L. *et al. Paenibacillus cymbidii* sp. nov., isolated from *Cymbidium goeringii* roots. *Int. J. Syst. Evol. Microbiol.* **71**, (2019).
30. Poehlein, A., Gottschalk, G. & Daniel, R. First Insights into the Genome of the Gram-Negative, Endospore-Forming Organism *Sporomusa ovata* Strain H1 DSM 2662. *Genome Announc.* **1**, e00734-13 (2013).
31. Lee, H., Chaudhary, D. K., Lim, O. B. & Kim, D.-U. *Paenibacillus agricola* sp. nov., isolated from agricultural soil. *Arch. Microbiol.* **205**, 248 (2023).
32. Jalal, M.A.F. & Collin, H.A. Estimation of caffeine, theophylline and theobromine inplant material. *New Phytol.* **76**, 277–281 (1976).
33. Aqel, A. *et al.* Rapid and Sensitive Determination of Methylxanthines in Commercial Brands of Tea Using Ultra-High-Performance Liquid Chromatography-Mass Spectrometry. *Int. J. Anal. Chem.* **2019**, 2926580 (2019).
34. Jeszka-Skowron, M., Frankowski, R., Zgoła-Grześkowiak, A. & Płatkiewicz, J. Comprehensive Analysis of Metabolites in Brews Prepared from Naturally and Technologically Treated Coffee Beans. *Antioxidants (Basel)* **12**, 95 (2022).
35. Mejía-Carmona, K. & Lanças, F. M. Modified graphene-silica as a sorbent for in-tube solid-phase microextraction coupled to liquid chromatography-tandem mass spectrometry. Determination of xanthines in coffee beverages. *J. Chromatogr. A* **1621**, 461089 (2020).
36. Thomas, J. B., Yen, J. H., Schantz, M. M., Porter, B. J. & Sharpless, K. E. Determination of caffeine, theobromine, and theophylline in standard reference material 2384, baking chocolate, using reversed-phase liquid chromatography. *J. Agric. Food. Chem.* **52**, 3259–3263 (2004).
37. Pomilio, A. B., Trajtemberg, S. & Vitale, A. A. High-performance capillary electrophoresis analysis of mate infusions prepared from stems and leaves of *Ilex paraguariensis* using automated micellar electrokinetic capillary chromatography. *Phytochem. Anal.* **13**, 235–241 (2002).
38. Wishart, D. S. *et al.* HMDB 5.0: the Human Metabolome Database for 2022. *Nucleic Acids Res.* **50**, D622–D631 (2022).
39. Willett, D. S., Filgueiras, C. C., Benda, N. D., Zhang, J. & Kenworthy, K. E. Sting nematodes modify metabolomic profiles of host plants. *Sci. Rep.***10**, 2212 (2020).
40. Kretschmar, J.A. & Baumann, T.W. Caffeine in Citrus flowers. *Phytochemistry* **52**, 19–23 (1999).
41. Brownstein, K. J., Tushingham, S., Damitio, W. J., Nguyen, T. & Gang, D. R. An Ancient Residue Metabolomics-Based Method to Distinguish Use of Closely Related Plant Species in Ancient Pipes. *Front. Mol. Biosci.* **7**, 133 (2020).

42. Afendi, F. M. *et al.* KNApSAcK family databases: integrated metabolite-plant species databases for multifaceted plant research. *Plant. Cell Physiol.* **53**, e1 (2012).
43. Xu, F., Gao, T. & Liu, X. Metabolomics Adaptation of Juvenile Pacific Abalone *Haliotis discus hannai* to Heat Stress. *Sci. Rep.* **10**, 6353 (2020).
44. Lampe, L., Jentzsch, M., Kierszniowska, S. & Levashina, E. A. Metabolic balancing by *miR-276* shapes the mosquito reproductive cycle and *Plasmodium falciparum* development. *Nat. Commun.* **10**, 5634 (2019).
45. Lai, X. *et al.* Integrated microbiome-metabolome-genome axis data of Laiwu and Lulai pigs. *Sci. Data* **10**, 280 (2023).
46. Han, X. *et al.* Disturbed microbiota-metabolites-immune interaction network is associated with olfactory dysfunction in patients with chronic rhinosinusitis. *Front. Immunol.* **14**, 1159112 (2023).
47. Yang, X. *et al.* Interaction between Cervical Microbiota and Host Gene Regulation in Caesarean Section Scar Diverticulum. *Microbiol. Spectr.* **10**, e0167622 (2022).
48. Li, L. *et al.* Gut microbiome drives individual memory variation in bumblebees. *Nat. Commun.* **12**, 6588 (2021).
49. Joffré, E. *et al.* Analysis of Growth Phases of Enterotoxigenic *Escherichia coli* Reveals a Distinct Transition Phase before Entry into Early Stationary Phase with Shifts in Tryptophan, Fucose, and Putrescine Metabolism and Degradation of Neurotransmitter Precursors. *Microbiol. Spectr.* **10**, e0175521 (2022).
50. Mashruwala, A. A. *et al.* The ClpCP Complex Modulates Respiratory Metabolism in *Staphylococcus aureus* and Is Regulated in a SrrAB-Dependent Manner. *J. Bacteriol.* **201**, e00188-19 (2019).
51. Steketee, P. C. *et al.* Transcriptional differentiation of *Trypanosoma brucei* during *in vitro* acquisition of resistance to acoziborole. *PLoS Negl. Trop. Dis.* **15**, e0009939 (2021).
52. Jeong, H. *et al.* Genome sequences of *Escherichia coli* B strains REL606 and BL21(DE3). *J. Mol. Biol.* **394**, 644–52 (2009).
53. Nelson, K. E. *et al.* Complete genome sequence and comparative analysis of the metabolically versatile *Pseudomonas putida* KT2440. *Environ. Microbiol.* **4**, 799–808 (2002).
54. Obranic, S., Babic, F. & Maravic-Vlahovicek, G. Improvement of pBBR1MCS plasmids, a very useful series of broad-host-range cloning vectors. *Plasmid* **70**, 263–7 (2013).
55. Rybtke, M. T. *et al.* Fluorescence-based reporter for gauging cyclic di-GMP levels in *Pseudomonas aeruginosa*. *Appl. Environ. Microbiol.* **78**, 5060–9 (2012).
