# Supplementary material for: Ubiquitous purine sensor modulates diverse signal transduction pathways in bacteria
Source: Nat Commun. 2024 Jul 12;15:5867. doi: 10.1038/s41467-024-50275-3 (PMC11245519; doi:10.1038/s41467-024-50275-3)
Supplement: Supplementary file 3 — Description of Additional Supplementary Files [file 41467_2024_50275_MOESM3_ESM.pdf]

Description of Additional Supplementary Files

Supplementary Data 1) Receptors that contain the dCache_1PU motif.

Supplementary Data 2) The isolation sources of bacterial strains that possess receptors with dCache_1PU domains.

Supplementary Data 3) Bayesian analyses settings.

Supplementary Data 4) AlphaFold2 model of McpH-LBD that was used to solve the crystallographic phase problem using Molecular replacement techniques.
