# Supplementary material for: Ubiquitous purine sensor modulates diverse signal transduction pathways in bacteria
Source: Nat Commun. 2024 Jul 12;15:5867. doi: 10.1038/s41467-024-50275-3 (PMC11245519; doi:10.1038/s41467-024-50275-3)

Isothermal Titration Calorimetry data for the analysis of purine binding to selected members of the dCache_1PU domain family

(R1) WP_219614703

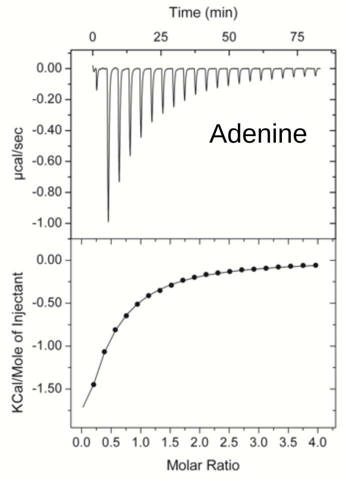

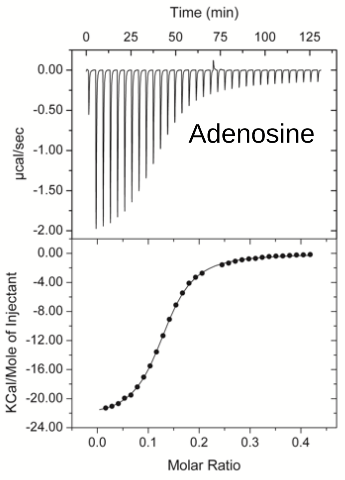

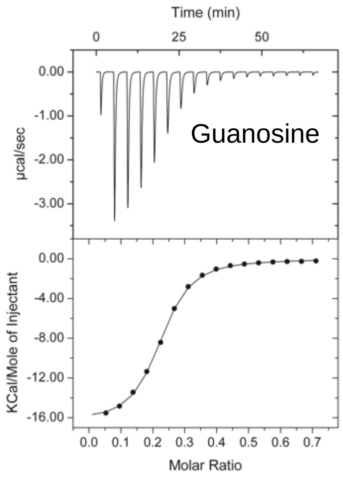

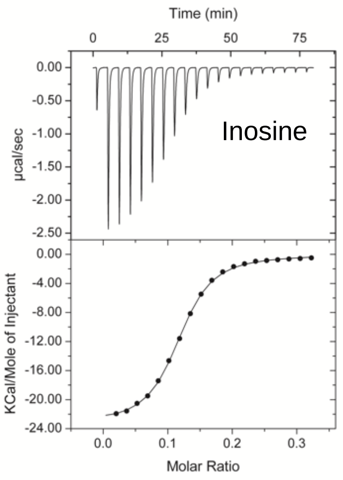

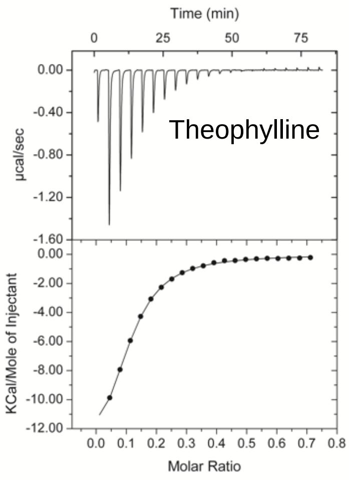

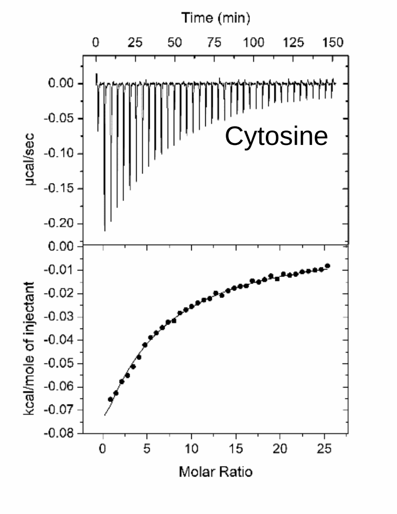

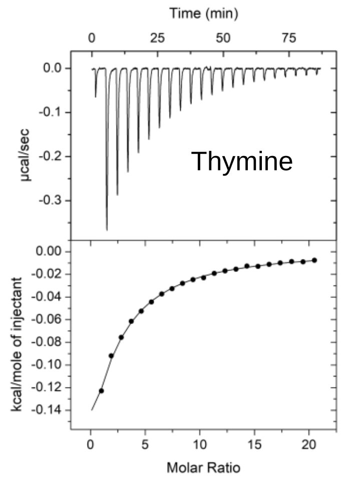

(R2) WP_107944080

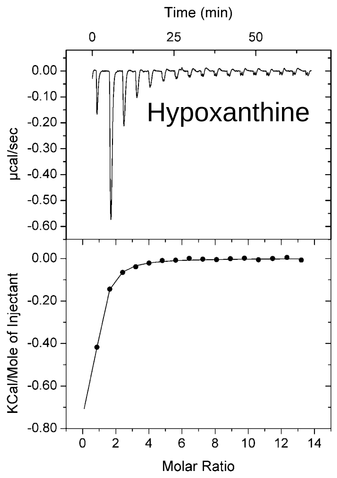

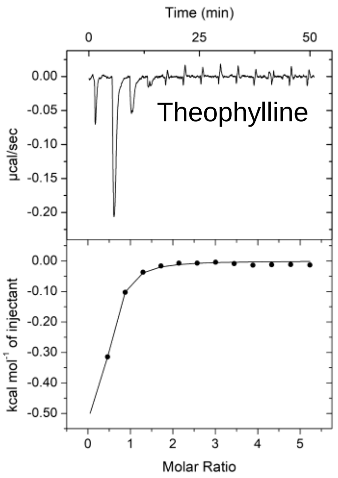

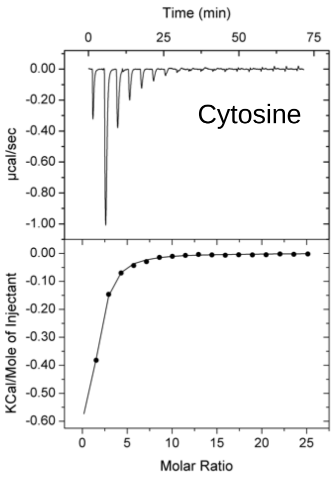

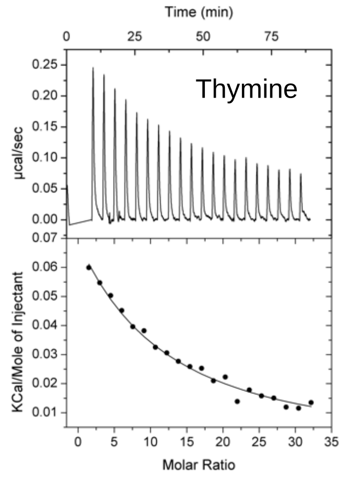

(R4) WP_199478134

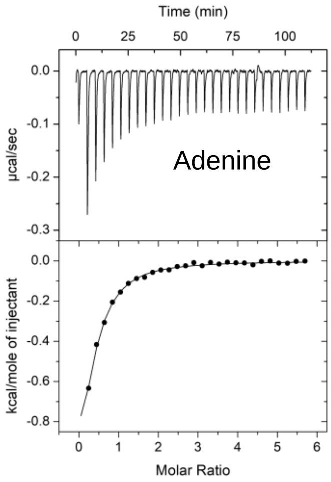

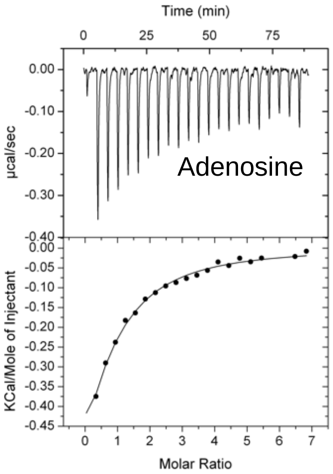

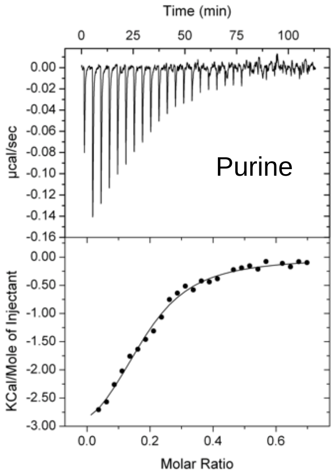

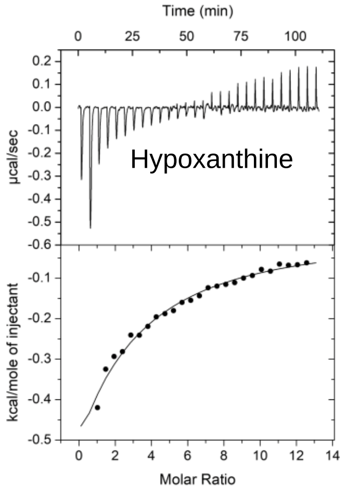

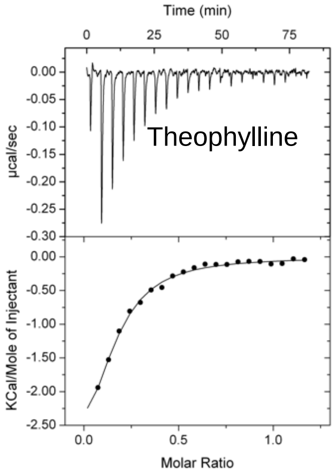

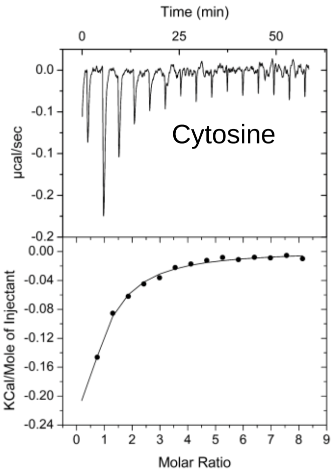

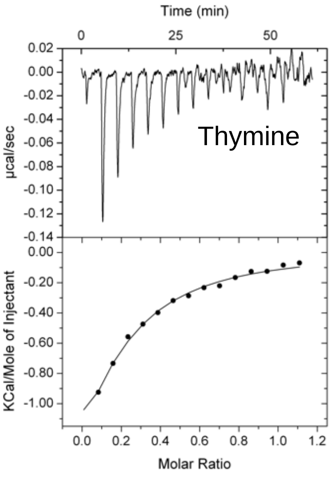

(R5) WP_131005693

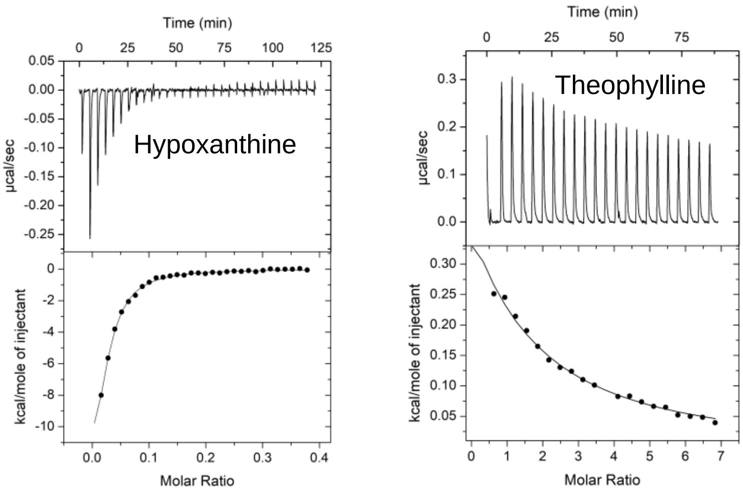

(R6) WP_185834821

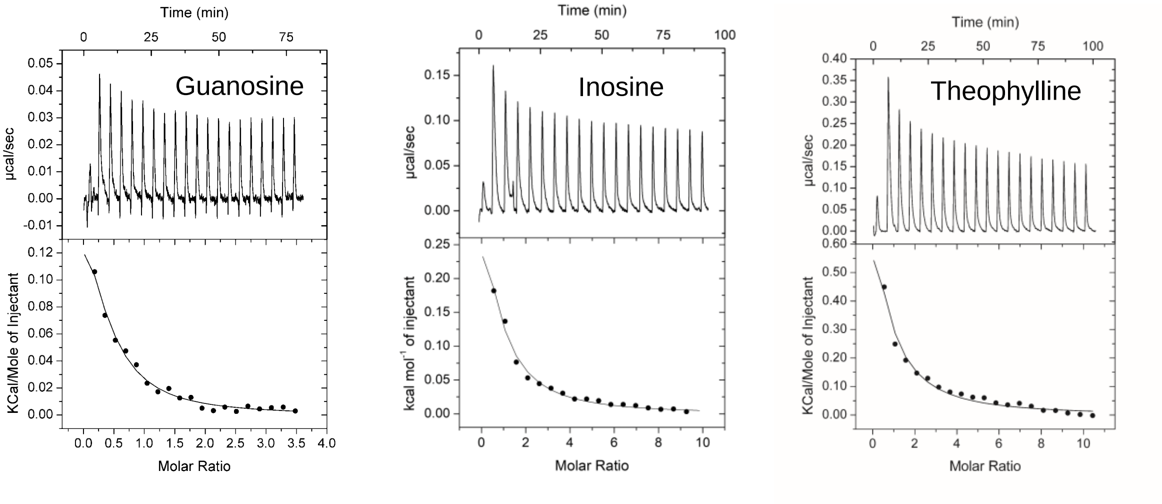

(R8) WP_227113344

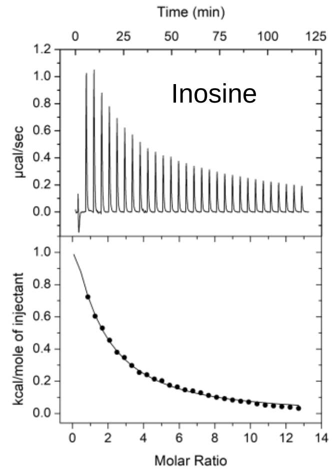

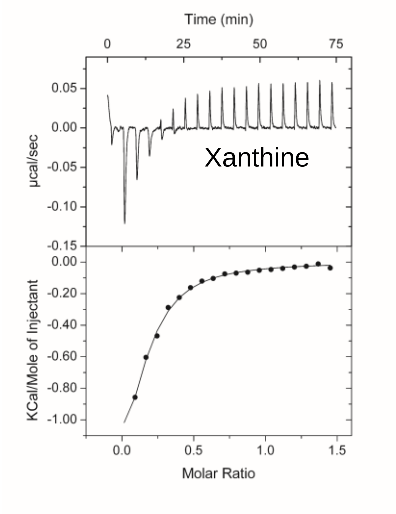

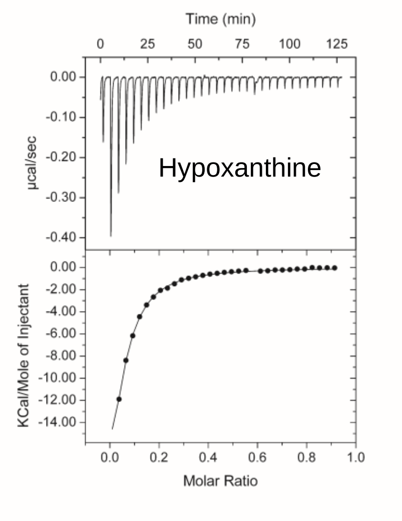

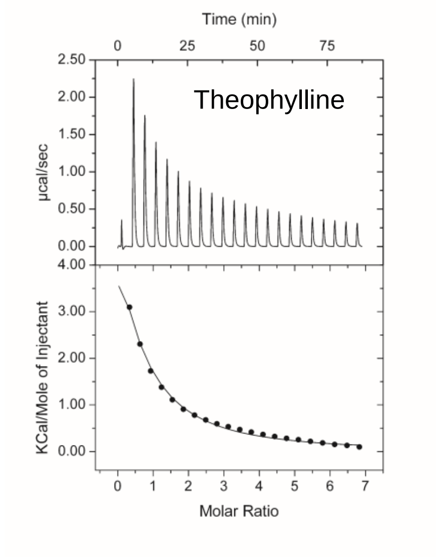

(R11) WP_162024566

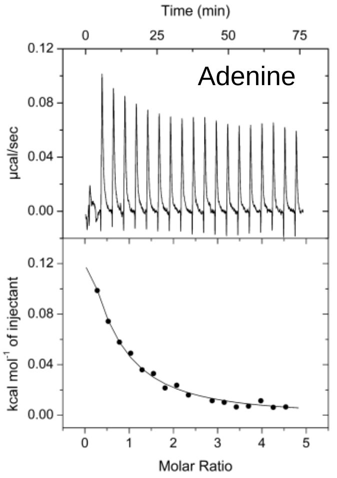

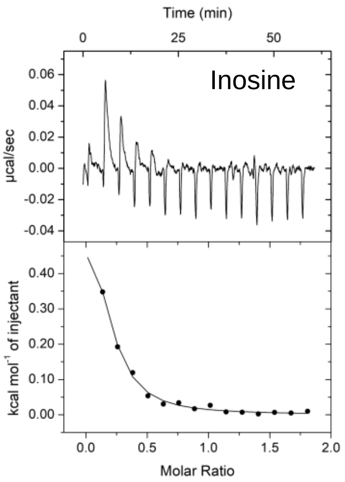

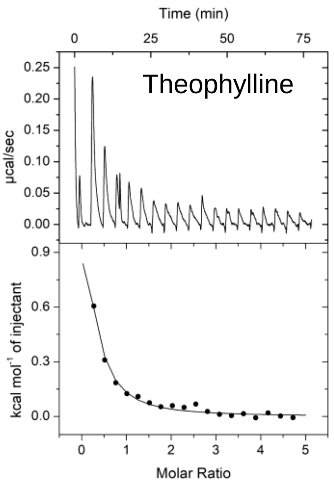

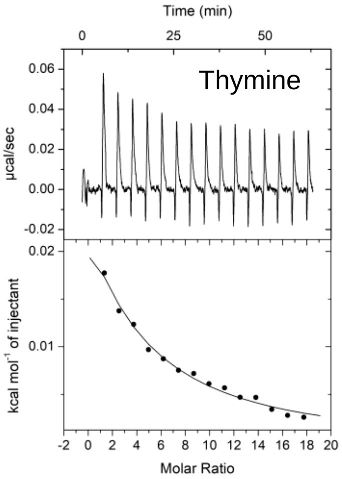

(R14) WP_021170906

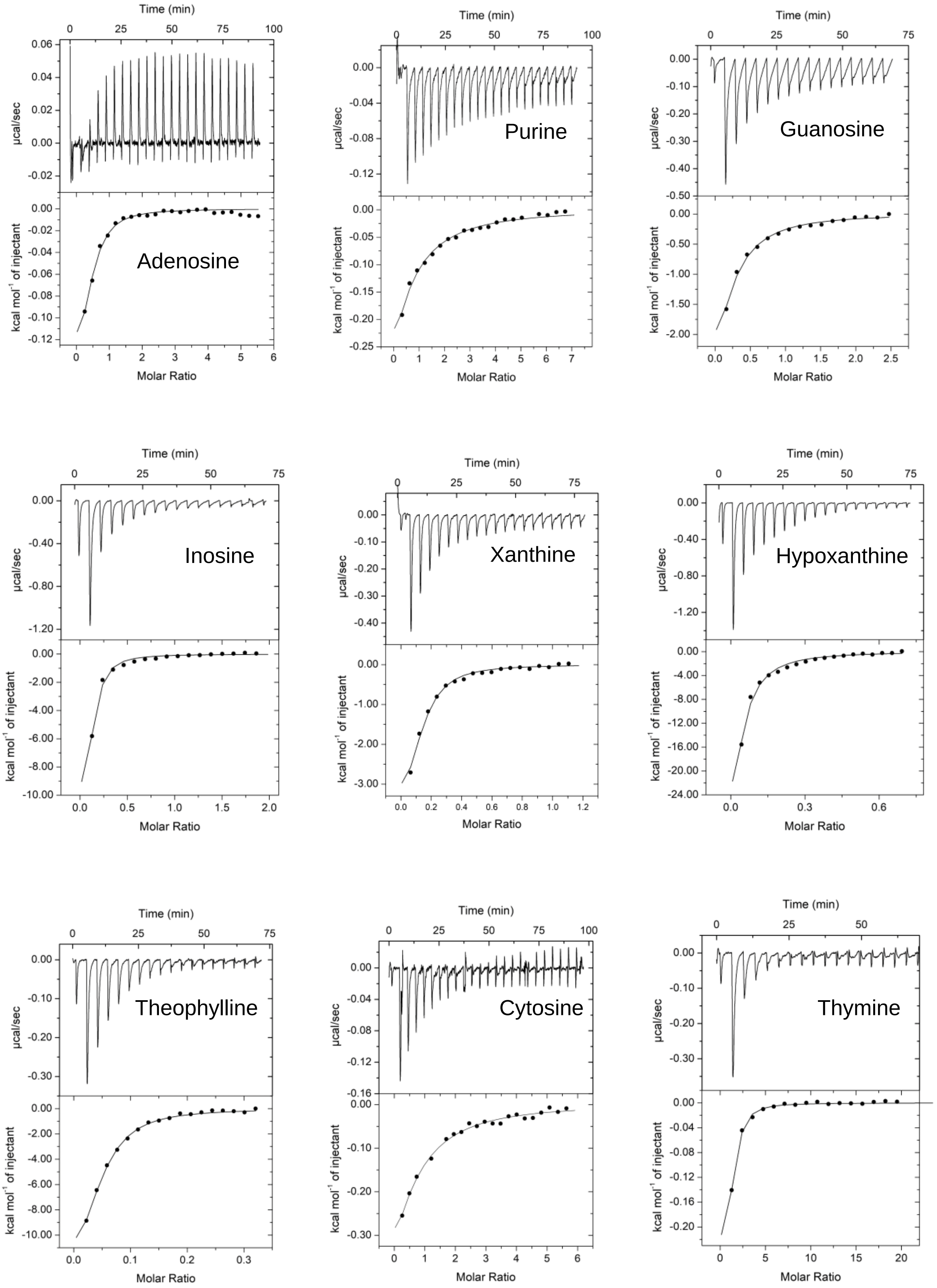

(R15) WP_207952809

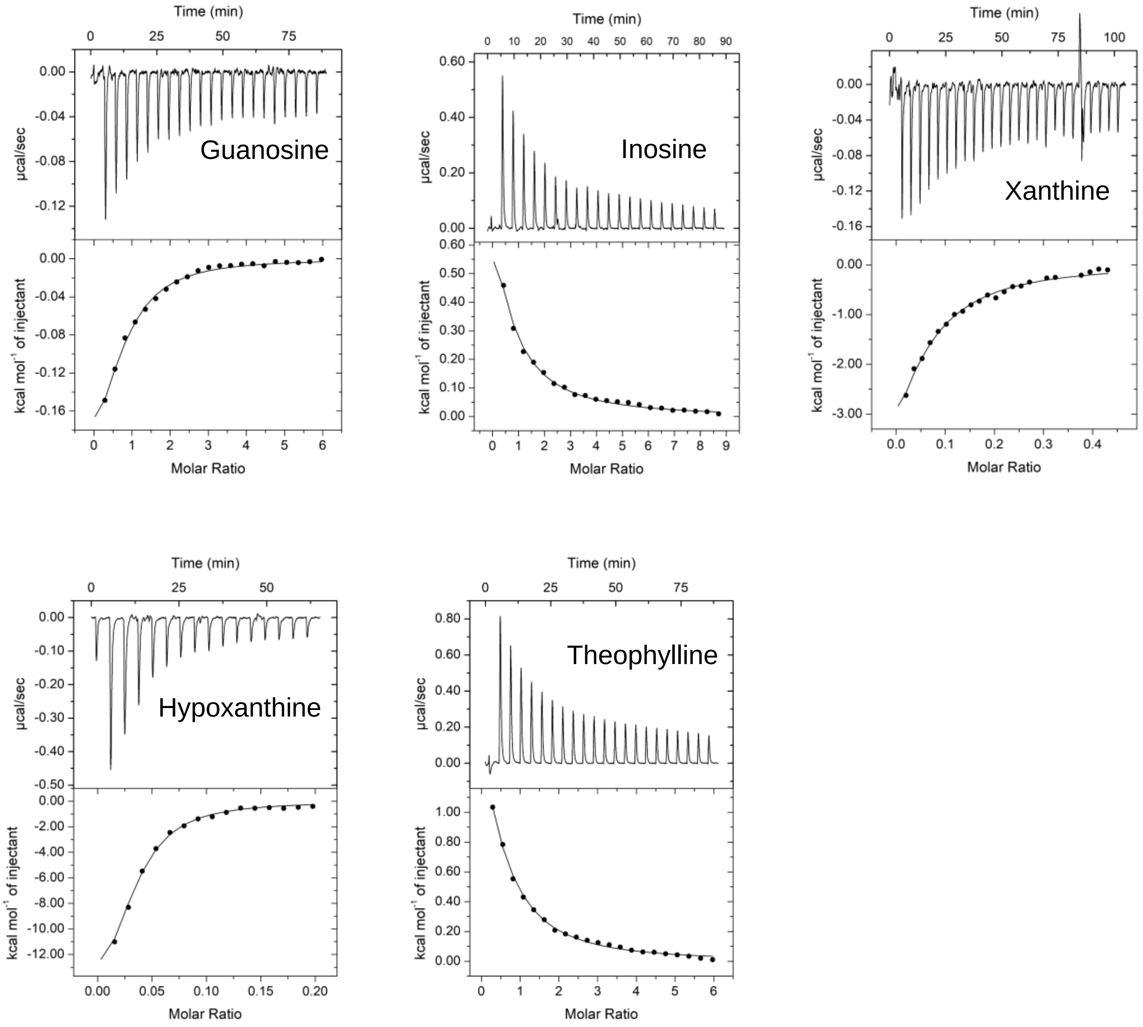

Supplement: Supplementary file 9 — Source Data [file 41467_2024_50275_MOESM9_ESM.zip › ITC figures (in Figshare).pdf]
